# Therapeutic efficacy of botanicals in psychological disorders in menopausal women: a systematic and scoping review

**DOI:** 10.3389/fphar.2025.1661035

**Published:** 2025-10-13

**Authors:** Arshiya Sultana, Md Belal Bin Heyat, Khaleequr Rahman, Zahoor Ahmed, Faijan Akhtar, Ansari Uzma Shamim, Khadija Khaleeq, Abdullah Y. Muaad

**Affiliations:** ^1^ Department of Gynecology and Obstetrics, National Institute of Unani Medicine, Ministry of Ayush, Bengaluru, India; ^2^ CenBRAIN Neurotech Center of Excellence, School of Engineering, Westlake University Hangzhou, Hangzhou, Zhejiang, China; ^3^ Department of Ilmul Saidla, National Institute of Unani Medicine, Ministry of Ayush, Bengaluru, Karnataka, India; ^4^ School of Medical Technology and Information Engineering, Zhejiang Chinese Medical University, Hangzhou, China; ^5^ School of Computer Science and Engineering, University of Electronic Science and Technology of China, Chengdu, China; ^6^ Rajarajeshwari Medical College and Hospital, MGR University, Bangalore, Karnataka, India; ^7^ IT Department, Sana’a Community College, Sana’a, Yemen

**Keywords:** menopausal women, depression, psychological symptoms, oxidative stress, herbal Unani medicine, female disorder, neurological disorder

## Abstract

**Introduction:**

Understanding the emotional impact of menopause on women is of utmost importance, especially with the predictable estimate of 1.2 billion menopausal women globally by 2030. Depression, anxiety, and stress are common during menopause, and botanical medicines, particularly from Unani traditions, may offer effective, natural therapeutic options. This study combines systematic and scoping review methods to assess the clinical efficacy of botanical interventions and map the broader research landscape.

**Methods:**

A comprehensive literature search was conducted across PubMed, Science Direct and PROSPERO from 2000 to 2024, following both PRISMA 2020 and PRISMA-ScR guidelines. The scoping phase identified a wide range of botanicals and research trends, while the systematic review focused on randomized controlled trials evaluating efficacy. Cochrane risk-of-bias tool was used for methodological quality assessment. Network visualization and word cloud techniques were also employed to identify related terms from the prior studies included in the analysis.

**Results:**

Sixteen RCTs involving 1,112 participants (mean age ±SD: 69.5 ± 21.88) were included. Most studies had a low risk of bias. Bioactive compounds such as withaferin A, quercetin, rosmarinic acid, and thymoquinone demonstrated antidepressant, anxiolytic, and neuroprotective effects through antioxidant, anti-inflammatory, GABAergic, and serotonergic mechanisms. Machine learning approaches showed potential for identifying compound interactions and personalizing treatment.

**Conclusion:**

Botanical medicines, especially from the Unani system, show promising efficacy in managing psychological symptoms during menopause. While current evidence is encouraging, further robust trials and mechanistic studies are needed. The integration of machine learning offers a novel direction for personalized phytotherapy.

**Systematic Review Registration:**

Identifier CRD42024514198.

## 1 Introduction

Menopause is a major life transition that marks the end of a woman’s reproductive years. It generally occurs between the ages of 45 and 55, characterized by a natural decline in estrogen and progesterone levels due to aging. The final menstrual period typically happens around the age of 51 (Kuck and Hogervorst, 2024). Menopausal symptoms vary widely among women and can include hot flushes, brain fog, night sweats, and sleep disturbances, impacting work productivity and daily life activities (Nappi et al., 2023). Women with severe menopausal symptoms often demonstrate higher levels of presenteeism and report facing greater workplace challenges compared to those without such symptoms (Whiteley J et al., 2013).

Studies have indicated that the likelihood of anxiety and depression in menopausal women is approximately 12.62% and 25.99%, respectively (Liu et al., 2023). Global studies show women are twice as likely as men to develop depression, especially during hormonal changes like postpartum and menopause. The SWAN MHS study found a threefold higher risk of major depression in late perimenopause and postmenopause compared to earlier stages (Chu et al., 2022). The relation between with menopausal symptoms and menopausal stages remains uncertain, with some attributing them to broader psychosocial factors like career and family issues rather than hormonal changes (Vanwesenbeeck et al., 2001). Several studies have identified diverse risk factors for depression during the menopausal transition. These include demographic factors (e.g., age, BMI, unemployment, financial stress), health history (especially prior depression), psychosocial stressors (e.g., life events, anxiety), hormonal changes (elevated FSH, LH, and estradiol), and menopausal symptoms like night sweats and vasomotor disturbances (Chu et al., 2022). Research indicates that higher rates of depression were observed in perimenopausal women (Bromberger et al., 2007). Besides in current years, several extensive community-based studies have indicated links between menopausal transition (MT) and heightened symptoms of depression, as well as an elevated risk of major depressive episodes However, some researchers contend that depression symptoms experienced during this phase might be attributed to life stress, sociodemographic factors, and deteriorating physical health. A longitudinal study revealed that the transition to menopause was associated with increased levels of anxiety among women who did not have anxiety during premenopause. Moreover, evidence suggests that among the anxiety symptoms studied, panic attacks may be more common during postmenopause (Mulhall et al., 2018). The economic impact of menopausal symptoms globally is substantial, estimated at $150 billion annually due to reduced work productivity, affecting one in three women during the menopausal transition (Kuck and Hogervorst, 2024). The clinical manifestations of the menopausal syndrome encompass a broad range of symptoms, including somatic symptoms, vasomotor, urogenital issues, sleep disturbances, mood changes, and sexual dysfunction. As a result, menopausal syndrome has a profound effect on women’s quality of life (Sultana et al., 2023).

Antidepressants and hormone therapy are commonly used to manage psychological symptoms associated with menopause. The use of Selective serotonin reuptake inhibitors (SSRIs) and serotonin-norepinephrine reuptake inhibitors (SNRIs) often limited due to side effects such as gastrointestinal issues, sexual dysfunction, weight gain, dizziness, and fatigue (Garay et al., 2019; Lopresti and Smith, 2021). Hormone therapy (HT) remains the primary treatment for menopausal symptoms, particularly in early perimenopause, but many women are unable or unwilling to use it due to associated risks, including endometrial and ovarian neoplasia, breast and uterine cancer, and increased risk of stroke. Non-hormonal alternatives like clonidine and gabapentin are also used but may lead to adverse effects such as sleep disturbances, nausea, constipation, and dizziness (Dehghan et al., 2022). These limitations highlight the growing interest in exploring safer, well-tolerated botanical therapies as potential alternatives for managing menopause-related psychological symptoms. Herbal medicine is estimated to be utilized by as many as four billion people, representing approximately 80% of the world’s population. Furthermore, around 22% of women in search of treatment for depression select to consult with a naturopath or herb doctor. Numerous herbal medicines and traditional Unani medicine have been individually investigated for their effectiveness in treating anxiety and depression (Clement et al., 2011). In recent years, a rising evidence has sustained the therapeutic potential of various botanicals in managing psychiatric conditions, including anxiety and depression. According to the guidelines developed by the World Federation of Societies of Biological Psychiatry (WFSBP) and the Canadian Network for Mood and Anxiety Treatments (CANMAT), several nutraceuticals and phytoceutical have shown encouraging efficacy in the treatment of mood and anxiety disorders (Sarris et al., 2022). Notable among these are *Hypericum perforatum* (St. John’s Wort), which has demonstrated efficacy comparable to standard antidepressants in mild to moderate depression (CANMAT, WFSBP Level 1 evidence). *Lavandula angustifolia* (Lavender) oil, particularly in its oral preparation (Silexan^®^), is recommended for generalized anxiety disorder (WFSBP Level 1 evidence). Additionally, *Crocus sativus* (Saffron) has shown antidepressant efficacy in randomized controlled trials and is considered a potential adjunctive treatment (CANMAT Level 2 evidence). *Rhodiola rosea* (Golden Root) has also been highlighted for its adaptogenic properties in reducing fatigue, stress, and mild depressive symptoms (CANMAT Level 2 evidence) (Ravindran et al., 2016). These botanicals represent promising alternatives or adjuncts to conventional pharmacotherapy, particularly for patients seeking integrative and holistic treatment approaches. These guidelines provide evidence-based references for clinicians, indicating that certain botanicals may be considered as adjunctive or alternative options, especially when conventional pharmacotherapy is contraindicated or not preferred. This growing recognition further supports the exploration of botanical medicines in addressing psychological symptoms during menopause (Sarris et al., 2022).

The Unani (Greco-Arabic) medicine is among the few ancient medical traditions that continue to thrive while preserving its classical foundations. Its core principles and theories remain relevant even in the present day. The term Unani Tibb or Unani Medicine reflects a rich historical legacy shaped and refined over centuries by diverse cultures, spanning regions from the Eastern Mediterranean and West Asia through North Africa, Hispano-Arabia, and Western Europe, to Central, South, and Southeast Asia (Sultana et al., 2015). The Greek philosopher and physician Hippocrates (460–377 BCE) familiarised the idea that disease arises from natural causes, and that its signs and symptoms are the body’s way of responding to illness. He is recognized as the originator of the humoral theory, which laid the foundation for later medical systems. It underscores the doctrine of the four elements, air, water, fire, and earth, and the four fundamental qualities (*Kayfiyat*): warm, cold, moist, and dry. Integral to its framework is the humoral theory, which describes four bodily humors blood, yellow bile, black bile, and phlegm. Health, according to this system, depends on the equilibrium among these humors, whereas any imbalance or excess leads to disease (Bhat et al., 2023). In Unani medicine, menopause (*Sinn-i-Inḥiṭāṭ or Sinn al-Yās*) is associated with a shift in *Mizāj* (temperament) toward *Burūdat* (coldness), leading to *Ihtibās al-Tamth* (amenorrhea) and reduced production of *Khilt Dam* (blood). This change contributes to symptoms such as headache, fatigue, anxiety, depression, weight gain, myalgia, and insomnia. Unani scholars attribute these to *Musharikat al-Raḥim* (uterine interaction) with other organs via *Bukhārat* (vapors) affecting the brain, heart, and musculoskeletal system (Sultana et al., 2023). Unani medicine emphasizes holistic health through lifestyle modification, dietary regulation, and the use of natural remedies, including plant-based formulations. It offers four principal modes of treatment: regimental therapy, diet therapy, pharmacotherapy, and surgery Among these, pharmacotherapy and regimental therapy play a key role in restoring the balance of humors to maintain overall health. First-degree drugs are considered the safest, possessing mild temperamental effects, while second-degree drugs are also regarded as safe but exhibit stronger temperamental properties without causing toxicity (Joonus Aynul Fazmiya et al., 2022). Unani botanicals are derived from medicinal plants recognized for their therapeutic properties and are used to restore humoral balance and improve physical and mental wellbeing. These botanicals are often prescribed based on individual temperament and symptomatology, aligning with the Unani philosophy of personalized care. Certain naturally occurring plant-based compounds have demonstrated beneficial effects in alleviating menopausal symptoms, offering effects comparable to hormone replacement therapy (HRT) but with fewer adverse outcomes. Notably, flavonoids and isoflavones phytoestrogens structurally similar to estrogen exert estrogenic activity in human tissues and are believed to offer protection against chronic conditions such as breast cancer, osteoporosis, and cardiovascular diseases. Additionally, these Unani botanicals exhibit emmenagogue, cardioprotective, anti-inflammatory, analgesic, and neuroprotective properties, making them valuable in managing menopausal symptoms (Sultana et al., 2023). Empirical studies analyzing Unani botanical effectiveness in this population are also on the rise. For example, in anxiety, Chamomile has shown significant reduction in symptoms and is well tolerated even at higher doses compared to placebo. Echinacea has demonstrated a decrease in anxiety over a short period of time (3 days). Passionflower has also shown promising results in reducing anxiety symptoms across multiple clinical trials. However, it is worth noting that many studies on herbal medicines for anxiety have been short-term, open-label, or inadequately reported (Casteleijn et al., 2019). In parallel, Unani medicine, a traditional system of healing, has been the focus of growing research interest for its potential in addressing mental health symptoms during menopause (Clement et al., 2011). Nevertheless, there is inconsistency in the data regarding the effectiveness of various herbal medicines in alleviating anxiety, depression, and stress among menopausal women. For example, some randomized controlled trials (RCTs) have reported significant improvements in psychological symptoms with the use of *Melissa officinalis* (Lemon Balm) (Shirazi et al., 2021), *Withania somnifera* (Ashwagandha), *C. sativus* (Lopresti and Smith, 2021)*, Nigella sativa* (Black seed oil) (Azami et al., 2022), *Matricaria chamomilla* (Bazrafshan et al., 2022), and multi-herbal formulations such as Aphrodit (a combination of *Zingiber officinale, C. sativus, Cinnamomum zeylanicum*, and *Tribulus terrestris*). Conversely, other studies have reported minimal or no benefit. These discrepancies may be attributed to variations in study design, sample size, population characteristics, dosage, duration of treatment, and outcome measures. Moreover, the use of different psychological assessment tools, such as the DASS-21, HADS, MRS, MKI and BDI further complicates cross-study comparisons (Table 1). These ongoing inconsistencies underscore the need for rigorous, standardized methodologies and comprehensive systematic evidence integration to clarify the true therapeutic potential of these botanicals. These ongoing controversies highlight the need for further clarification through systematic evidence integration.

**TABLE 1:** The published RCTs on Unani Botanicals.

S. No.	Scientific/Unani/Common name	Study/Blinding/RCT/Control	Participants	Age (Y)	Intervention and sample size	Control	Duration of treatment (weeks)	Tool	Menopausal status	Main results	Side Effect/ADR reported or not reported	Ref.
1	*Withania somnifera* Dunal/*Asgandh/*winter Cherry	Single-blind Placebo	45	45–55	6 g twice daily powder (n = 30)	Placebo (Wheat flour) (n = 15)	12	HAS PSQIDURAT for insomnia, hot flashes and night sweat	Postmenopausal patients	Significant change in HAS	Reported	Banu and Begum (2012)
2	*Withania Somnifera* Dunal	Double-blind, Placebo	100	-	300 mg root extract twice daily (n = 50)	Placebo (n = 50)	8	MRS, MENQoL, S. estradiol, FSH and LH and testosterone	Perimenopausal women with climacteric symptoms	Signicant difference in the psychological, somato-vegetative and urogenital serum estradiol, FSH and LH in experimental group	Not reported	Gopal et al. (2021)
3	*Melissa officinalis* L./*Badranjboya*/Lemon Balm	Three arm, Double-Blind Standard and Placebo Control	60	*Postmenopasual*	500 mg daily (n = 20)	Placebo (n = 20) and citalopram (30 mg) (n = 20)	8	Pittsburg Sleep quality index MENQQOL	Postmenopausal women with sleep disorder	Improvement in vasomotor symptoms sleep disorders, psychomotor-social, and sexual domains in the experimental group	Reported	Shirazi et al. (2021)
4	*Nigella sativa* L. oil/*Shuneez*/Black seeds	Triple-Blind, Placebo	72	40–60	*N. sativa* oil capsule (1000 mg) (n = 36)	placebo capsule (n = 36)	8	Greene’s Climacteric Scale	Menopausal women	Remarkable decrease in Greene’s Climacteric Scale	Reported	Azami et al. (2022)
5	*Tribulus terrestris* L./*Khar-e-khask*/Puncture vine	Single-blind, RCT, Placebo	60	≥35	3 g powder of Tribulus twice daily (n = 30)	Placebo (n = 30)	8	MRS	Women with perimenopausal symptoms	Significant decrease in MRS scores in the test group	Reported	Fatima and Sultana (2017)
6	*Glycyrrhiza glabra* L	Single-blind Placebo	40	41–55	2 g powder in capsules of *G. glabra* twice daily (n = 20)	Roasted wheat flour (n = 20)	8	MRS	Menopausal women complaining of postmenopausal symptoms	Reduction in MRS score and improvement of postmenopausal symptoms was noted	Not reported	Rais et al. (2020)
7	*Foeniculum Vulgare* Mill/*Badiyan*/Fennel seeds	Triple-blind Placebo	71	45–60	Fennel seed powder (2 g) and daily (n = 36)	Starch (n = 35)	8	MKI and s estradiol	Post menopausal women with changes in sexual desire (45–60 years)	Fennel seed significantly improved menopausal symptoms	Not reported	Ghaffari et al. (2020)
8	*Tribulus terrestris, Zingiber officinale, Crocus sativus* extract, and *Cinnamomum zeylanicum*/Aphrodit (ginger, saffron, cinnamon, and Tribulus terrestris)	Triple-blind, Placebo	80	50–60	Capsule contained 40 mg of *T. terrestris,* 12.27 mg of *Z. officinale*, 3 mg of *C. sativus* extract, and 11 mg of *C. zeylanicum* (n = 40)	Placebo capsules contained 50 mg of starch (n = 40)	4	MRS	80 postmenopausal women self-reported menopause symptoms	Aphrodit was effective in ameliorating menopausal symptoms	Reported	Taavoni et al. (2017)
9	*Zingiber officinalis* Roscoe/*Zanjabeel*/ginger	Double-blind Placebo	50	45–60	*Z. officinale* (1000 mg) (n = 30)	Starch (n = 20)	12	MRS, S.estradiol, FSH, LH, Progesterone	Menopausal women, and	*Z. officinale* significantly reduced the intensity of menopausal symptoms with a significant change in estrogen and FSH levels (p < 0.001)	Not reported	Taha and Dizaye (2022)
10	*Crocus sativus* L./*Zafran*/Saffron	Double-blind, Placebo	86	40–60	14 mg of a standardized saffron extract (n = 43)	Placebo (n = 43)	12	GCS, PANAS SF-36	Perimenopausal women experiencing menopausal complaints	Significantly reduction in the GCS psychological score from baseline to week 12 in test group	Reported	Lopresti and Smith (2021)
11	*Crocus sativus* L	Double-blind, Placebo	60	>40	Saffron 30 mg/day in two divided doses (n = 30)	Placebo (n = 30)	6	HFRDIS HDRS score	post-menopausal women with hot flashes and depression	Significant effect for time × treatment interaction on the HFRDIS score HDRS score [F (3, 162) = 5.48, p = 0.001]	Reported	Kashani et al. (2018)
12	*Matricaria chamomilla/Lavandula officinalis* L./Lavender and Chamomile Herbal Tea	Single- blind Three arm, Placebo	96	>45	2 g of dried lavender (n = 32) or chamomile leaves (n = 32), which were cooked twice daily once in the morning and once at night—in 300 mL of boiling water for 10–15 mt and consume two cups	Placebo (n = 32)	2	BDS SAS	Postmenopausal women with anxiety and depression	Drinking lavender or chamomile herbal tea could alleviate the level of anxiety and depression in postmenopausal women.	Not reported	Bazrafshan et al. (2022)
13	*Vitex agnus-castus/Sambhalu*/Vitex	Double-blind, Placebo	52	45–65	30-mg Vitex (n = 26)	placebo (n = 26)	8	GCQ	postmenopausal women	The mean scores for total menopausal disorder, anxiety, and vasomotor dysfunction were significantly lower in the Vitex group after treatment	Reported	Naseri et al. (2019)
14	*Pimpinella anisum* L./Anisoon/Aniseed	Double-blind placebo	60	40–60	3 daily capsules (667 mg each) of anise (n = 30)	Placebo (n = 30)	8	DASS, MKI	Women with menopausal symptoms	*P. anisum* significantly reduced the DASS-21 and MKI	Reported	Begum and Sultana (2023)
15	Mixed herbal medicine (Fennel, Chamomile, and Saffron)	Triple-blind, Four arm	120	45–65	four groups: A (250 mg chamomile, 30 mg fennel, 15 mg saffron), B (1000 mg, 120 mg, 60 mg), and D (500 mg, 60 mg, 30 mg)	placebo ^©^ (n = 30)	12	MRS	Women with menopausal symptoms	A 12 weeks extracts treatment, there were significant improvement in physical, psychological and urogenital domains in group B	Not reported	Mahdavian et al. (2019)
16	*Saliva officinalis* extract/Sage	Blinding not mentioned	60	46–58	100 mg of dry extract of sage (n = 30) one tablet	Placebo (n = 30)	4	MRS	Postmenopausal with Menopausal symptoms	Severity of hot flashes, night sweats, panic, fatigue, and concentration had significant differences before and after the consumption of sage extract	Not reported	Dadfar and Bamdad (2019)

To address this gap, this study adopts a dual approach by combining both a systematic review and a scoping review. The systematic review component focuses specifically on evaluating the clinical efficacy and safety of Unani botanicals in alleviating depression, anxiety, and stress in menopausal women, using data from RCTs and formal risk-of-bias assessments. The scoping review component aims to map the breadth of existing research on botanical interventions, identify commonly studied herbs, network visualization and literature mapping, detect research gaps and propose future research directions for integrative, evidence-based mental health interventions in menopause care. Given the wide heterogeneity in study designs, interventions, and outcome measures, the scoping review would provide essential insights that informed and refined the focus of the systematic review. This hybrid approach allows for a comprehensive understanding of the field both in terms of evidence quality and research direction.

The specific research questions guiding the study. These include:

1. What is the clinical efficacy of Unani and other botanical medicines in alleviating depression, anxiety, and stress in menopausal women, based on RCTs evidence?
2. What are the main bioactive compounds and their proposed mechanisms of action?
3. What gaps exist in the current research, and how might future directions, including machine learning applications, enhance understanding and treatment?

## 2 Materials and methods

### 2.1 Review design

This review was designed as a hybrid coalescing both a systematic review and a scoping review approach to assess the efficacy and safety of Unani botanicals in alleviating psychological symptoms (depression, anxiety, and stress) in menopausal women. This study was conducted in two phases: an initial systematic review followed by a scoping review. The systematic component included the identification and critical appraisal of RCTs, concentrating on clinical evidence, whereas the scoping component facilitated to explore broader aspects such as mechanisms of action of herbal compounds and emerging computational methods. The scoping review was undertaken to explore the extent, range, mechanism of action and nature of research activity related to herbal interventions for psychological symptoms in menopausal women. The methodology followed PRISMA 2020 guidelines for systematic reviews and the PRISMA-ScR extension for scoping reviews, along with referencing the CONSORT statement [22] for the evaluation of trial quality. Visual tools, including word clouds to visually represent key concepts [23] and network visualization techniques based on keyword analysis, were employed to identify thematic clusters and conceptual relationships between studies.

A thorough methodology is methodically outlined, incorporating diverse data collection and analysis procedures. The study adheres to PRISMA standards for randomized controlled trials (Bin Heyat et al., 2021; Heyat et al., 2021; Page et al., 2021; Teelhawod et al., 2021; Akhtar et al., 2022) and utilizes a guideline checklist (Hussain et al., 2018) to ensure methodological rigor. Key steps in the methodology include (a) preparation of protocol to register in PROSPERO, (b) developing clear selection criteria, (c) a comprehensive literature search from on-line database and grey literature, (c) Data extraction includes screening of titles, abstracts, and keywords, (d) Analysing and synthesizing the results and information (Abelha et al., 2020; Singh and Kumar, 2020), (e) risk bias and quality assessment.

### 2.2 Protocol registration

The protocol was officially registered at PROSPERO, University of New York, under the registration number CRD42024514198, and version 1.1 published on 29 February 2024 and version 1.2 was updated on 28 July 2025. The scoping review component was not part of the original registration. However, it was added as an amendment during the manuscript writing phase of study execution to map the broad evidence base. This amendment has been transparently reported in accordance with PRISMA-ScR extension and PRISMA 2020 guidelines.

### 2.3 Eligibility criteria

The inclusion criteria were defined as follows: participants of menopausal age experiencing psychological symptoms diagnosed by scales such as DASS-21, Hamilton anxiety scale, menopause rating scale (MRS), or other diagnostic scales for menopausal symptoms, and receiving either Unani botanicals/herbal medicine in RCTs. Interventions were administered orally for at least two cycles, with control interventions including placebo or Western medicine. Clinical trials with a minimum intervention duration of 8 weeks were considered. Full-text access and only English language articles was required for validation purposes. There were no restrictions on publication status, and dissertation/grey literature was also included. Exclusion criteria comprised patients with known psychiatric disorders, severe systemic diseases, pregnancy or lactation, and studies utilizing routes of administration other than oral. Only human studies were considered, with quasi-RCTs, non-RCTs, case series, conference manuscript, editorials, posters, and unreliable data excluded. Irrelevant studies and those lacking sufficient quantitative data were excluded. Studies with inadequate outcome reporting or lacking validated psychological assessment tools were also omitted. In some cases, the botanical interventions were either not clearly defined or did not align with the traditional Unani criteria for inclusion. Additionally, studies involving populations outside the menopausal age range or not specifically targeting menopausal women were also excluded. The selection process was conducted in accordance with the PRISMA and CONSORT reporting guidelines to ensure transparency and completeness of reporting. Study quality and methodological reliability were assessed using Cochrane risk of bias tools, and duplicate records were identified and removed prior to screening.

The scoping review, this study included primary studies, reviews, and other relevant literature focusing on herbal or botanical interventions for anxiety, depression, and stress in menopausal women.

Furthermore, the plants botanical names were verified according to the World Flora Online (http://www.worldfloraonline.org, accessed on 30 December 2024). Data from animal experiments and *in vitro* studies were utilized to explore the etiopathogenesis of psychological symptoms in menopausal women and the pharmacological effects of herbs. Ultimately, studies were considered reliable and incorporated if they met established criteria. All collected data underwent review and evaluation by the researchers, followed by additional individual checks conducted by other authors.

### 2.4 Information sources and search strategies

In this review, the researchers searched electronic databases including PubMed and Science Direct to retrieve primary studies published between 2000 and 2024 on psychological symptoms in menopausal women. Also, PROSPERO was accessed to identify current or ongoing systematic reviews on related topics.

To ensure an adequate number of relevant studies, the search strategy included the PICOS framework (Population, Intervention, Comparator, Outcomes, and Study design), Boolean operators (AND/OR) and Medical Subject Headings (MeSH) were used to refine the search to include RCTs on herbal treatments for psychological symptoms in menopausal women. The following combination of keywords and phrases were applied during the search process: (“Unani medicine” OR “herbal medicine” OR “herbal supplement” OR “botanical”) AND (“menopause” OR “postmenopausal”) AND (“depression” OR “anxiety” OR “stress” OR “psychological symptoms”), (“Herbs” AND “menopausal symptoms”) OR (“depression and menopause”) OR (“anxiety and menopause”), (“RCT” OR “randomized controlled trial”) AND (“DASS” OR “Hamilton anxiety scale” OR “HADS”), (“antioxidants” OR “anti-inflammatory”) AND (“herbs” OR “botanicals”). Filters were applied to limit results to human studies and articles published in only English language. No restrictions were placed on study location or participant ethnicity.

The Rayyan online software (https://www.rayyan.ai, accessed from 24 April 2024) was used to manage the selection process. Firstly, it was employed to identify and manage duplicates within the collected publications, ensuring that each unique study was considered only once during the review process. Secondly, Rayyan also facilitated blinded screening of titles and abstracts by two independent reviewers and streamlined exclusion based on pre-defined inclusion/exclusion criteria. Only RCTs that examined the efficacy of herbal or Unani treatments for psychological symptoms in menopausal women were included. Lastly, the software was utilized to streamline the exclusion process by assisting in the removal of publications that did not meet the eligibility criteria outlined for the study. Overall, Rayyan served as a valuable tool for enhancing the efficiency and rigor of the article selection process.

### 2.5 Selection process and data extraction

**FIGURE 1:**
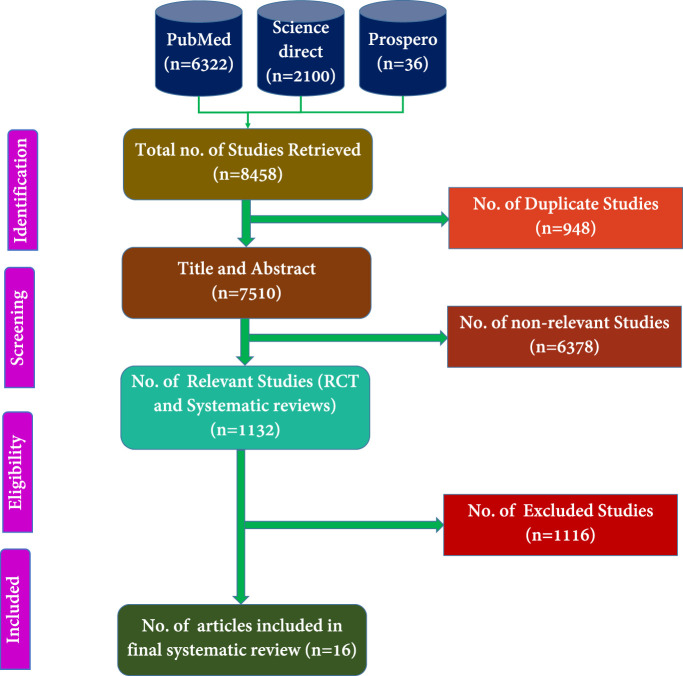
PRISMA of the proposed study.

The selection process involved an initial screening of titles, abstracts, and keywords to determine article eligibility, followed by full-text reviews. Three independent reviewers screened all retrieved titles and abstracts. Eligible full-texts were reviewed in accordance with PRISMA and CONSORT guidelines. Any disagreements were resolved through consensus with a fourth and fifth reviewer. The selection process was visually presented using a PRISMA flow diagram (Figure 1). Final inclusion decisions were made for articles published between 2000 and 2024.

Comprehensive records were maintained, capturing details such as authors, sample size, participant demographics, study design, data collection methods, randomization procedures, blinding techniques, intervention specifics, intervention duration, measured outcomes, pharmacological characteristics, phytoconstituents, and reported adverse effects in data extraction. Disagreements were resolved through cross-validation by a fourth and fifth reviewer. Studies lacking sufficient quantitative data or those deemed extraneous were excluded.

Figure 1 presents the total number of articles identified from the PubMed, PROSPERO, and Science Direct databases. The subsequent steps involved selecting RCTs for systematic review and excluding irrelevant studies. Dissertation works were also incorporated into the analysis. These selected articles were then subjected to additional evaluation based on their abstracts, titles, and keywords, in line with the exclusion criteria established.

### 2.6 Risk of bias (RoB) and quality assessment (QA)

First reviewer, conducted assessments of the risk of bias and the quality assessment of the included published articles. The Cochrane Risk of Bias Tool version 1.0 assesses the methodological quality of randomized controlled trials across seven key domains. These include random sequence generation and allocation concealment, both of which address selection bias. Performance bias is evaluated through the blinding of participants and personnel, while detection bias is assessed via blinding of outcome assessment. Attrition bias is examined through the handling of incomplete outcome data, and reporting bias is considered by assessing selective outcome reporting. The final domain addresses other potential sources of bias not captured in the previous domains, such as early trial termination or baseline imbalances. Each domain is rated as having a low, high, or unclear risk of bias, providing a structured approach to evaluating the internal validity of included studies. Any discrepancies that arose were resolved through consensus among all the authors. Any inconsistencies in the data were rectified through mutual agreement (Sultana et al., 2022).

### 2.7 Synthesis of results

Both quantitative and qualitative approaches were followed for synthesis of results. For the systematic review, findings from RCTs were organized into summary tables and analyzed based on the change in psychological symptom scores before and after intervention. Where possible, percentage improvements and comparative effect sizes were calculated. For the scoping review, a thematic synthesis was performed, grouping studies by bioactive components, pharmacological effects, and herbal mechanisms of action such as antioxidant, anti-inflammatory, MAO-inhibitory, and serotonergic pathways. Network visualization and word cloud techniques were applied to identify frequently occurring terms and conceptual links between studies. This dual-synthesis strategy enabled a comprehensive interpretation of the evidence base, identification of research gaps, and recommendations for future directions in the clinical application of Unani herbal treatments for menopausal psychological symptoms.

## 3 Results and Discussion

### 3.1 Selection of source of evidence and AMSTAR-2 criteria

The manuscript has been enhanced by incorporating essential methodological elements guided by the AMSTAR-2 checklist to improve its completeness and quality. As part of the methodological rigor, the study adhered to key domains outlined in the AMSTAR-2 (A MeaSurement Tool to Assess Systematic Reviews) checklist. A clear research question was formulated using the PICO framework, and a comprehensive literature search was performed across multiple databases with defined inclusion and exclusion criteria. The selection of studies was conducted independently by two reviewers, with any disagreements resolved through consensus. The risk of bias in included studies was assessed using appropriate tools, and a transparent description of the data extraction and synthesis methods was provided. Additionally, funding sources and conflicts of interest for the included studies were reported where available.

We retrieved 8,458 titles and abstracts, from which 948 duplicates were identified and removed using Rayyan online software. Among the remaining 7,510 articles, 1,132 were relevant to RCTs conducted on menopausal women, while 6,378 were deemed irrelevant. The 1,132 potentially relevant articles were screened, and 72 full-text RCTs were evaluated for eligibility. After further review, 16 full-length RCTs that met the inclusion criteria were selected for the systematic review and data extraction phase (Figure 1).

Several types of studies were excluded from the present review based on predefined eligibility criteria. Studies involving populations other than naturally menopausal women, such as post-hysterectomy or surgically induced menopause or only one symptoms, were excluded; for example, Valerian root on hot flashes in menopausal women (Mirabi and Mojab, 2013). Trials addressing non-psychological menopausal symptoms like vasomotor or somatic or sexual issues without evaluating psychological outcomes were also omitted such as the use of fennel vaginal cream or *Apium graveolen*’s fruits (Abedi et al., 2018; Hessami et al., 2021), aromatherapy (Abbaspoor et al., 2022) and studies including exercise for vasomotor symptoms were excluded (Thomas and Daley, 2020). Lastly, non-original research articles including reviews, editorials, and case reports such as the narrative review by Kennedy (2016), (Yazdanpanahi et al., 2016) was also not included. These exclusions ensured that only relevant, high-quality clinical studies directly assessing botanical interventions in psychological disorders among menopausal women were synthesized.

### 3.2 Characteristics of included sources of evidence

A total of 16 RCTs involving 1112 participants (mean ± SD: 69.5 ± 21.88; variance: 548.44) were identified. Among these, two studies involved a combination of herbal medicines, while the remaining 14 focused on single herbal medicines for managing menopausal symptoms. Additionally, two dissertation works were included. Other indexing sources were not considered, ensuring that the research questions addressed most relevant published articles. Table 1 Provides detailed characteristics of previously published studies including study design, participant numbers, tools (DASS-21, MRS), intervention types, and outcomes related to herbal medicines.

**FIGURE 2:**
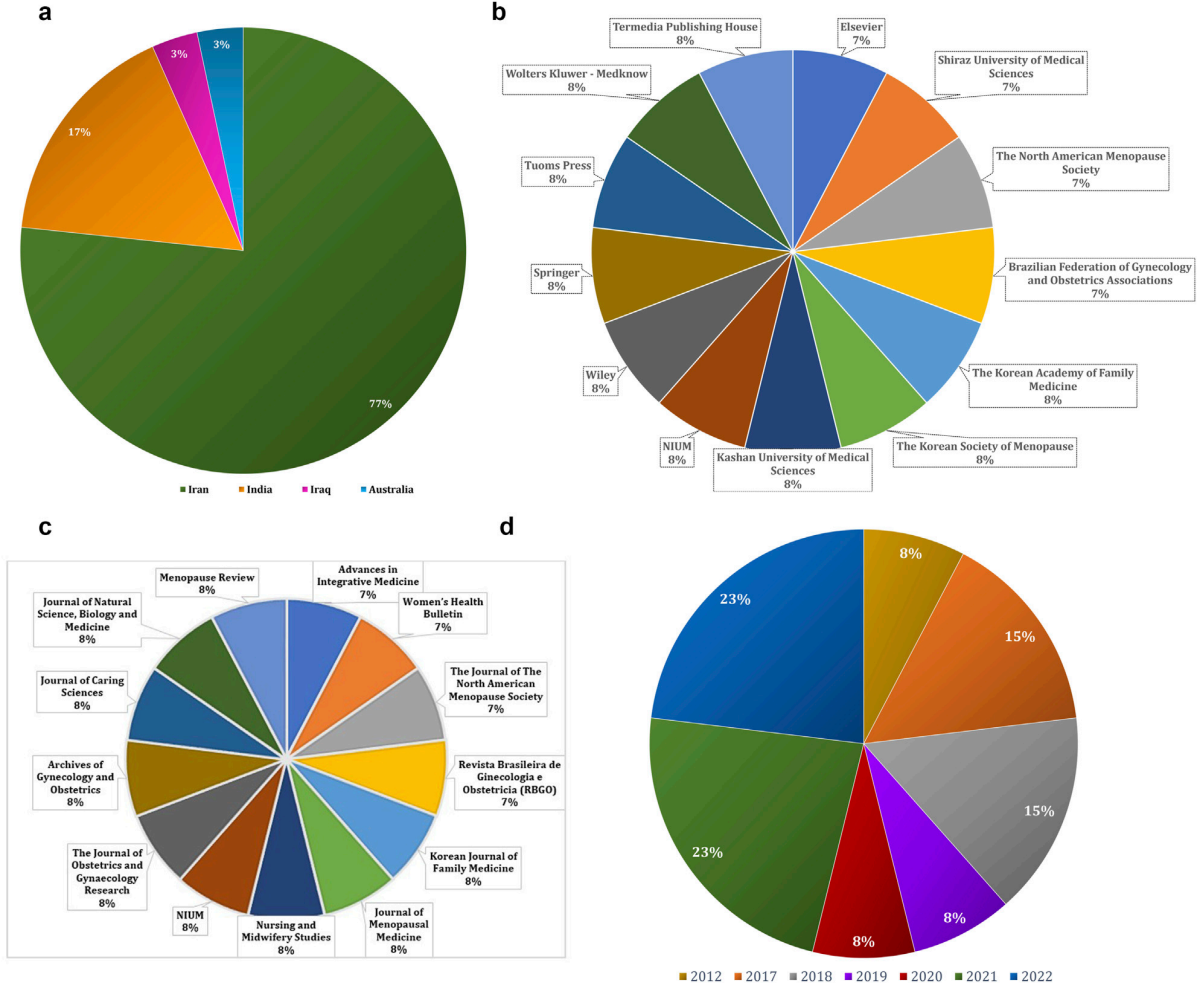
**(a)** Country-wise **(b)** Publisher-wise **(c)** Journal-wise and **(d)** Year-wise previously published articles on psychological menopausal symptoms and herbal medicine.

The present data as per country, publishers, and Journals sources shared in the publications related to the menopausal symptoms in women. Geographically, most studies were conducted in Iran, which accounted for approximately 77% of the publications, followed by India (17%), Iraq (3%), and Australia (3%) (Figure 2a). Regarding journal sources and publishers, the majority of publications were clustered among a few leading publishers, as shown in Figures 2b,c. Year-wise distribution revealed that research output increased notably in 2021 and 2022, which together represented 46% of total studies, followed by 2018 (18%) (Figure 2d). In addition to peer-reviewed articles, two dissertations were also included in the analysis to provide broader coverage of the literature.

### 3.3 Critical appraisal within sources of evidence

**FIGURE 3:**
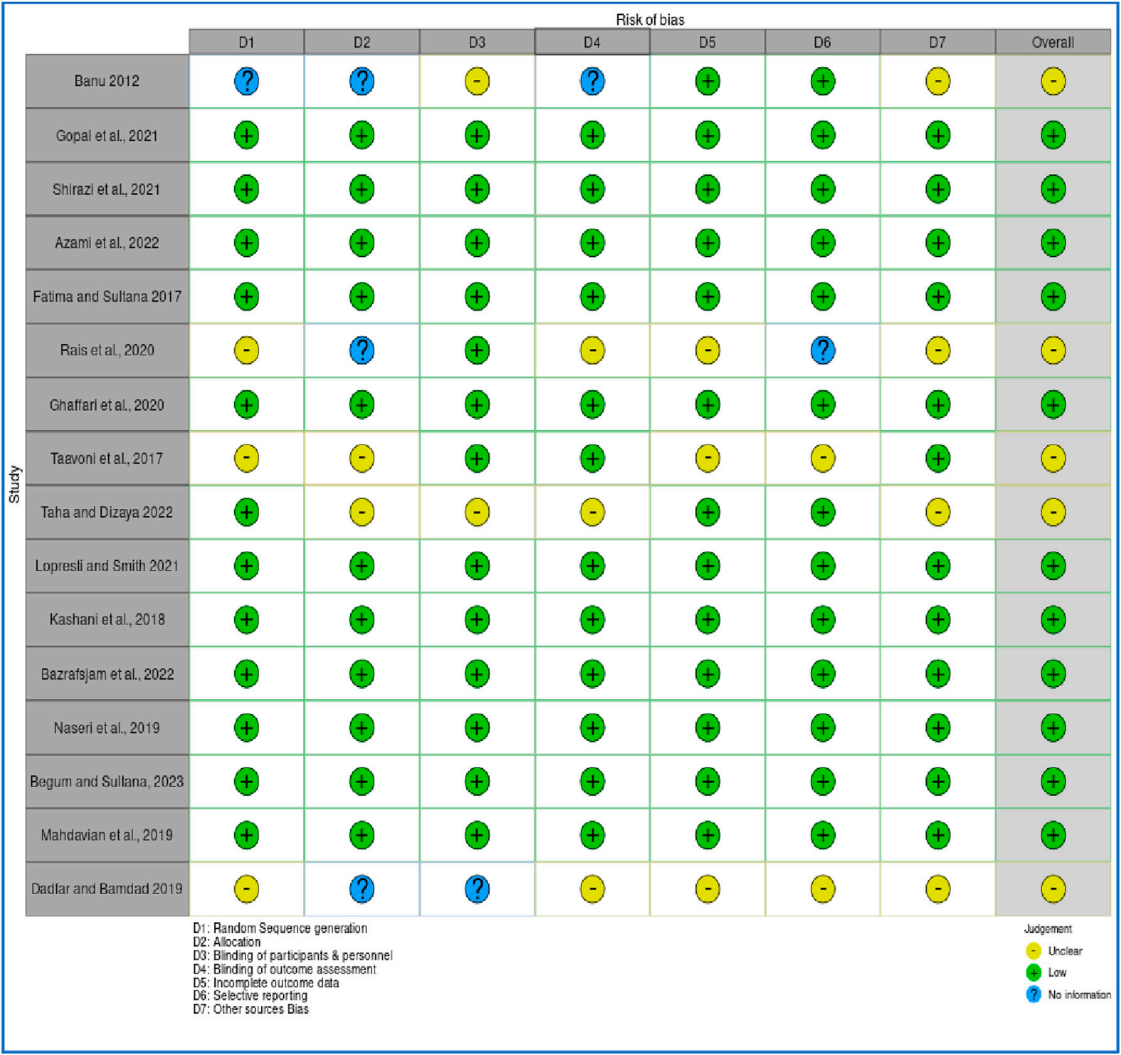
Quality assessment of randomized controlled trials.

**FIGURE 4:**
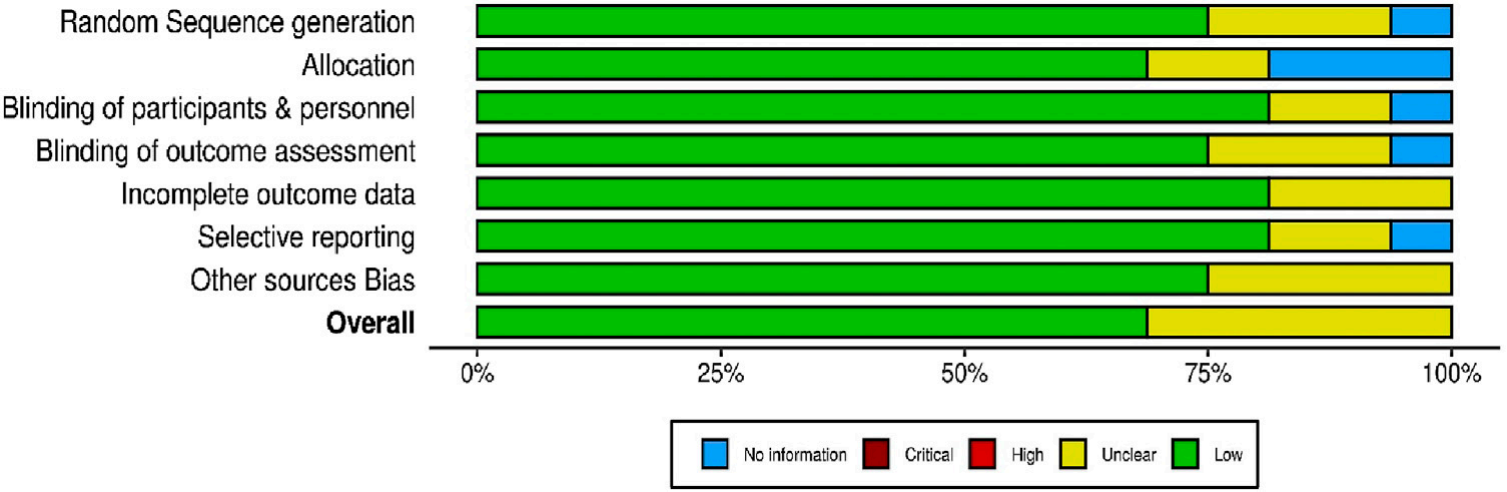
Summary plot of randomized controlled trials.

The methodological quality and risk of bias of the 16 included randomized controlled trials (RCTs) were evaluated using a modified version of the Cochrane Risk of Bias (RoB 1.0) tool, adapted for assessing randomized studies. To visualize the results, the Robvis web application https://mcguinlu.shinyapps.io/robvis/was utilized on 26 December 2024, generating both a traffic light plot (Figure 3) that illustrates individual study-level assessments and a summary plot (Figure 4) that aggregates domain-level judgments across all studies. Most studies were found to have a low to moderate risk of bias, suggesting an overall acceptable level of methodological quality and reliability in the included evidence. Specifically, several trials including those by Azami et al., Bazrafsham et al., Gopal et al., Shirazi et al., and Naser et al. exhibited low risk in key domains such as random sequence generation, allocation concealment, handling of incomplete outcome data, and selective reporting. Nevertheless, certain studies showed areas of concern. For example, insufficient reporting of blinding procedures for participants, personnel, and outcome assessors led to an unclear risk of performance and detection bias in studies by Taavoni et al. (2017), Taha and Dizaye (2022), and Mahdavian et al. (2019). Furthermore, inadequate details on randomization methods and allocation concealment were observed in studies such as Banu and Begum, (2012), Dadfar and Bamdad (2019), and Rais et al. (2020), further contributing to an unclear risk assessment. Overall, while the majority of the RCTs demonstrated sound methodological practices, limitations in the reporting of blinding and randomization processes in a few studies should be taken into account when interpreting the overall findings of this scoping review.

### 3.4 Results of individual sources of evidence

Each included study was systematically monitored to extract relevant data aligning with the review objectives. These data included intervention details (e.g., type of herbal medicine, dosage, duration), participant characteristics, baseline scores of depression and anxiety, and post-intervention outcomes. Many studies reported statistically significant improvements in depression and anxiety scores compared to placebo or conventional treatments, particularly with herbs like *Withania somnifera, Melissa officinalis*, and *Nigella sativa*. Additionally, studies often reported minimal or no adverse effects, supporting the safety profile of Unani and traditional botanicals.

### 3.5 Synthesis of results

The synthesis combined quantitative and thematic analyses. Quantitatively, the 16 RCTs exhibited a consistent trend toward improvement in psychological symptoms among menopausal women using herbal interventions. Mean symptom reduction scores were calculated where available, though heterogeneity in outcome measures precluded a formal meta-analysis.

Thematic analysis underscored common pharmacological mechanisms across the herbal treatments, such as antioxidant, GABAergic, anti-inflammatory, and serotonergic activity. Word cloud analysis, and Network visualization supplementary revealed frequently used terms and concepts related to herbal treatment mechanisms and study focus areas. Common bioactive compounds recognized across studies included withaferin A, quercetin, thymoquinone, rosmarinic acid, and ursolic acid, suggesting potential pathways for clinical efficacy.

The inclusion of systematic as well as scoping review elements facilitated a multidimensional understanding of the evidence. The systematic component delivered robust evaluation of RCTs and the scoping review endorsed exploration of broader questions, such as mechanisms of action and publication trends. This comprehensive synthesis provides a foundation for future research, particularly in guiding clinical applications and identifying areas requiring further investigation.

### 3.6 Major findings of this review

To the best of our review, this is the first systematic review to assess the effectiveness of Unani botanicals in depression, anxiety and stress symptoms in menopausal women. Table 1 highlights RCTs examining Unani botanicals for managing psychological menopausal symptoms. The study formulated research questions addressing the efficacy of Unani botanicals in depression, anxiety, and stress in menopausal women and emphasizing the role of Unani botanicals and their bioactive components. Computational tools were applied for network mapping, and word cloud creation, facilitating insights into these questions and guiding future research on menopausal symptom management. In 16 studies most of the research showed significant improvement in psychological symptoms in menopausal women. All studies were randomized, with four employing single-blind methods, four using triple-blind designs, seven implementing double-blind protocols, and one study not specifying its blinding approach. In all studies, the comparator was placebo. Most studies were two-arm, except for three. Shirazi et al. used a three-arm design, which included standard citalopram, *M. officinalis*, and a placebo group. Bazrafshan et al. also conducted a three-arm lavender, chamomile, and placebo study. Mahdavian et al. used a four-arm design, with different chamomile, fennel, and saffron doses, along with a placebo group. Of the 16 studies, nine reported side or adverse effects. Most studies provided insights into the mechanisms and pharmacological properties of the herbal medicines evaluated. In eight studies, the duration of intervention was 8 weeks. The tools used for diagnosing psychological menopausal symptoms included HAS, MRS, Greene’s Climacteric Scale, MKI, HDRS, BDS, SAS, and DASS. The MRS psychological subscale was used in seven studies, while DASS was used in only one study.

### 3.7 Pathophysiology of psychological symptoms (depression, anxiety and stress) in menopause

In this section we provide a comprehensive analysis of the mechanisms underlying perimenopausal psychological symptoms including depression, anxiety and stress, emphasizing the intricate connections between hormonal changes, neurotransmitter function, BNDF, chronic inflammation, and oxidative stress.

The menopausal transition endocrinology is highly complex and exhibits significant variation among women. The decline in ovarian follicle numbers, caused by atresia or ovulation, forms the foundation of reproductive aging, a process that unfolds throughout a woman’s life. The complicated feedback loop between the ovaries and the HPA remains a challenging aspect of understanding reproductive endocrinology. Gonadotropins play a central role in regulating the secretion of ovarian steroid hormones (estradiol, progesterone, and testosterone) and inhibins A and B. Inhibin B levels are closely linked to the number of developing ovarian follicles, while AMH, produced by granulosa cells in the ovaries, functions independently of gonadotropin regulation (O’Neill and Eden, 2017).

During the initial menopausal transition period, the follicle numbers declines and it becomes critical. This leads to a reduction in follicular phase inhibin B levels and a subsequent rise in follicle-stimulating hormone (FSH). The rise in FSH levels is due to a reduction in inhibin B production by the antral follicles. This disruption in the feedback mechanism is complex, leading to a shift from primarily regular ovulatory cycles to mainly irregular or anovulatory cycles as the final menstrual period (FMP) nears. Although the number of follicles decreases during this transition, increased FSH continues to stimulate the remaining ovarian follicles, helping sustain normal serum E2 levels until the later stages of the menopausal transition (O’Neill and Eden, 2017).

Fluctuations in pituitary gonadotropins and AMH play a role in intermittent ovulation and irregular cycle lengths, which characterize the menopausal transition. Changes in cycle length are a key clinical marker of this phase, though a significant number of women experience minimal alterations. While testosterone levels remain relatively stable during this period, DHEAS levels gradually decrease with advancing age (O’Neill and Eden, 2017).

Estrogen plays a key role in perimenopausal depression by regulating neural circuits, including serotonin, noradrenergic, and dopaminergic systems. Its neuroprotective effects have been observed in conditions like schizophrenia, Alzheimer’s disease, and Parkinson’s disease (Liang et al., 2024). Clinical trials indicate that discontinuing hormone therapy in women previously responsive to it can trigger a recurrence of depressive symptoms (Schmidt et al., 2015). Additionally, estrogen supplementation has been shown to alleviate physical and depressive symptoms in perimenopausal patients. These findings suggest that declining estrogen levels during perimenopause weaken its neuroprotective effects, contributing to depressive symptoms (Liang et al., 2024).

The menopause transition is widely recognized as a pivotal period in women’s lives, marked by an increased risk of mental health challenges. Earlier theories attributed this heightened risk to grief over the loss of fertility or depressive moods stemming from “empty nest syndrome”. This contemporary framework highlights the critical role of hormonal changes during menopause, their direct effects on the brain, and their contribution to distressing menopausal symptoms such as hot flashes, sleep disturbances, vaginal dryness, and cognitive issues, which collectively diminish quality of life (Gordon et al., 2021).

**FIGURE 5:**
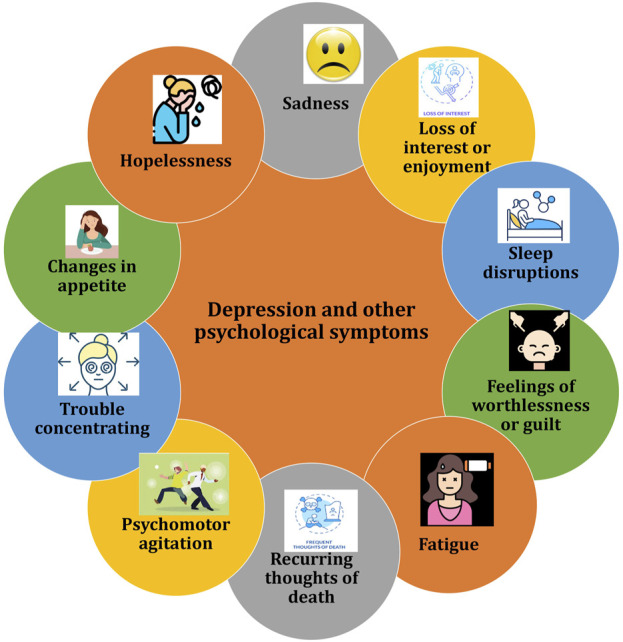
Illustration of common symptoms associated with depression and other psychological symptoms.

Depression encompasses various mood, cognitive, and behavioral symptoms that can cause significant distress and impair daily functioning. Common symptoms include persistent sadness and hopelessness and other depicted in Figure 5 (Liang et al., 2024). With the rapid increase in the global elderly population, particularly among women undergoing menopause, perimenopausal depression poses a significant healthcare and financial burden (Liang et al., 2024). Perimenopausal depression is marked by emotional disturbances including anxiety, depression and stress, often related to endocrine imbalances like hypogonadism and aging. Current treatment options mainly aim to relieve symptoms but are often accompanied by adverse effects. Advances in creating therapies that target the underlying mechanisms of perimenopausal depression have been slow. Fluctuations in estrogen and progesterone levels during perimenopause are linked to a heightened risk of depression, as these hormonal changes may trigger increased production of proinflammatory mediators and oxidative stress, potentially leading to gradual neuronal damage (Liang et al., 2024).

Weber et al. (2014) in their a systematic review highlighted a higher risk of depressive symptoms and depressive disorders during the tmenopausal stages compared to the premenopausal phase Similarly, Hickey et al. (2016) observed a consistent trajectory of depressive symptoms in the menopausal transition period, independent of vasomotor symptoms. Their findings, based on a 15-year cohort study involving 13,715 women with age between 45 and 50 (ALSWH), indicated elevated depressive symptoms in perimenopausal women (Hickey et al., 2016). Another systematic review and meta-analysis examined the risk of clinical depression in menopausal stages. Seventeen prospective cohort studies (n = 16,061) were reviewed, with meta-analyses including seven studies (n = 9,141). Using random-effects models, perimenopausal women showed a significantly higher risk of depressive symptoms and diagnoses compared to premenopausal. However, no significant increase in risk was found for postmenopausal women. Limitations include variability in menopausal stage classifications and depression measures, contributing to heterogeneity. A direct comparison of peri- and post-menopause was not possible due to limited longitudinal data (Badawy et al., 2024).

The monoamine hypothesis links depression to reduced levels of norepinephrine, serotonin, and dopamine in the CNS, supported by the efficacy of MAOIs and TCAs in increasing these neurotransmitters. Reduced plasma tryptophan and cerebrospinal serotonin levels further support this theory, forming the basis for SSRIs. Estradiol influences serotonin regulation by enhancing 5-HT2A receptor expression, reducing serotonin breakdown via lower MAO-A levels, and modulating tryptophan hydroxylase gene expression. Brain imaging shows elevated MAO-A levels in perimenopausal women, correlating changes in gonadal hormones with serotonin deficiency and depression risk (Rekkas et al., 2014; Hernández-Hernández et al., 2018; Moncrieff et al., 2023).

Decreased BDNF levels in the hippocampus and prefrontal cortex are linked to estrogen deficiency and contribute to depression in perimenopausal women. Estrogen increases BDNF through estrogen receptor β, promoting cell survival and synaptic plasticity via signaling pathways like PI3K/Akt, MEK/ERK, and mTORC1. This pathway also regulates autophagy and nerve regeneration. BDNF plays a role in the HPA axis and serotonin system, helping alleviate stress-induced depression. Clinical studies indicate that inflammatory markers are positively associated with depression scores, while BDNF levels show an inverse relationship with these scores and are connected to estradiol levels in perimenopausal women (Scharfman and MacLusky, 2006; Hui et al., 2016).

The hyperactivity of the HPA axis is linked to anxiety, depression, and cognitive dysfunction. Stress or perceived threats activate the HPA axis, involving the amygdala and paraventricular nucleus (PVN), which triggers the release of neuropeptides like CRH and AVP. These peptides stimulate the pituitary gland to release ACTH, which prompts the adrenal glands to release glucocorticoids and mineralocorticoids. These hormones circulate through the bloodstream and cerebrospinal fluid, where they bind to steroid receptors and play a role in regulating stress responses (Gjerstad et al., 2018). The HPA and HPG axes are closely related and interact in estrogen-related affective disorders. Estrogen influences the synthesis and secretion of hormones through the HPG axis and plays a crucial role in the onset and progression of female MDD (Liang et al., 2024). For instance, contraceptive use can affect women’s emotional experiences. Estrogen also enhances the effectiveness of SSRIs, improving treatment outcomes for perimenopausal depression. Studies show that estrogen antagonists increase stress responses, while low-dose estradiol in ovariectomized mice reduces stress-induced ACTH release, suggesting that estrogen modulates the HPA axis to prevent its overactivation (Zanardi et al., 2007) (Figure 5).

### 3.8 Role of neuroinflammation and oxidative stress in depression associated with menopause

Two key factors in perimenopause, bioenergetic deficits and chronic inflammation, are linked to genetic risk factors for neurodegenerative diseases and MDD. Pro-inflammatory cytokines (IL-8 and TNF-α) are upregulated in perimenopausal women, with estradiol levels inversely related to these cytokines and neuroinflammation. Estrogen decline activates immune cells, increasing pro-inflammatory cytokines like IL-6, IL-1, and TNF-α. These cytokines can reach the brain via leaky circumventricular regions, active transport, endothelial activation, nerve fiber signaling, and microglial involvement. IL-1β, expressed in the hippocampus, may impair memory functions through dysregulated cytokine signaling. Pro-inflammatory cytokines also affect neurotransmitter responses, influencing glutamate and GABA systems (Garlanda et al., 2013; Lee et al., 2022). Research has demonstrated that inflammatory cytokines such as IL-6, IL-1β, and TNF-α can disrupt neuronal function by diminishing BDNF the neuroprotective effects. IL-1β inhibits the activation of the TrkB receptor and disrupts its associated downstream signaling pathways such as PLCγ1 and the MAPK pathway, though it does not significantly affect TrkB expression. Elevated IL-1β levels beyond the physiological range upregulate p38 MAPK, disrupting BDNF-dependent synaptic plasticity. Inhibiting p38 MAPK can prevent these harmful effects on neural plasticity (Tong et al., 2012).

Oxidative stress happens when the production of reactive oxygen species (ROS) or reactive nitrogen species (RNS) exceeds the body’s ability to neutralize and eliminate them and leading to cellular damage. In major depressive disorder (MDD), excessive ROS contribute to neuronal damage, impacting mood regulation and neurodegenerative processes. Brain tissue, rich in lipids and with high oxygen consumption, is particularly vulnerable to ROS-induced damage, which can harm lipids, DNA, and proteins. ROS, such as superoxide anions (O_2_^−^), can also interact with nitric oxide (NO) to form peroxynitrite, impairing enzyme functions and reducing the synthesis of key neurotransmitters. Additionally, ROS can trigger the neurotoxic kynurenine pathway, further harming neurons. Mitochondria, the main source of ROS, generate superoxide during electron transport, and inflammation can exacerbate this process, creating a vicious cycle of ROS production and neuroinflammation. To counter this, cells produce antioxidant molecules like glutathione peroxidases, catalase, and superoxide dismutase, regulated by the Nrf2 pathway. Under normal conditions, Nrf2 is degraded by Keap1, but oxidative stress activates Nrf2, enhancing antioxidant enzyme production. Nrf2 activators, such as melatonin or rice protein, have shown promise in alleviating depressive behaviors by reducing oxidative stress. Furthermore, cyclic GMP (cGMP), synthesized in response to nitric oxide, plays a role in regulating oxidative stress and neuroinflammation. Estrogen can increase cGMP levels, and postmenopausal women experience a decline in both estrogen and cGMP, leading to heightened oxidative stress. Clinical studies have observed that postmenopausal women exhibit higher levels of oxidative stress compared to premenopausal women, accompanied by reduced levels of antioxidant enzymes. Estrogen replacement therapy has been shown to help reduce oxidative stress by enhancing the expression of antioxidant genes, suggesting its potential as a therapeutic approach for managing oxidative stress-related conditions, including depression (Mor et al., 2021; Hassamal, 2023; Simpson and Oliver, 2020).

**FIGURE 6:**
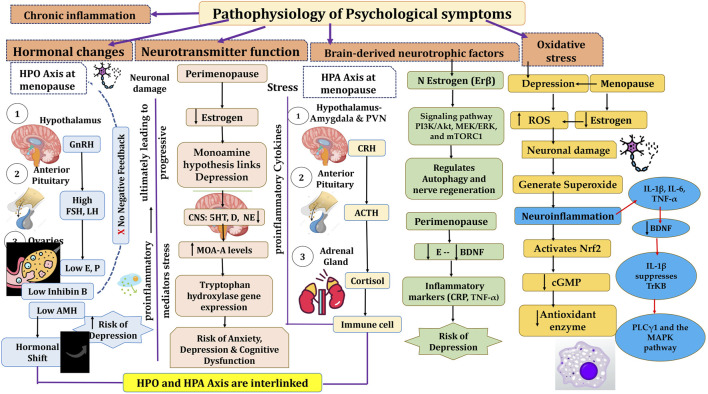
Pathophysiology of menopause and psychological symptoms associated with menopause. HPO, Hypothalamic pituitary ovarian; GnRH, Gonadotrophic Releasing Hormone; FSH, Follicle stimulating hormone; LH, Luteinizing Hormone; HPA, Hypothalamic pituitary Adrenal; CRH, Corticotrophic Releasing Hormone; ACTH, Adrenocorticotrophic Hormone; BDNF, Brain Derived Neurotrophic Factor; ROS, Reactive Oxygen Species; IL, Interleukins; TNF, Tumor Necrosing Factor.

Notably, depressive symptoms are most prevalent during early perimenopause, making this stage a crucial window for implementing effective treatments compared to later menopausal phase. Advancing research into the underlying mechanisms of perimenopausal depression is critical for detecting therapeutic targets and developing effective, targeted treatment approaches (Liang et al., 2024) (Figure 6).

### 3.9 Mechanisms of action of bioactive molecules of Unani botanicals in psychological symptoms in menopause

Perimenopausal depression is commonly treated with conventional antidepressants, including tricyclic antidepressants, monoamine oxidase, and selective serotonin reuptake inhibitors (Garay et al., 2019). Estrogen therapy are also used to manage symptoms and slow disease progression. Recently, newer pharmacological options, including SNRIs, glutamatergic agents, and SERMs, have gained attention for their improved efficacy and reduced side effects (Littleton-Kearney et al., 2002).

**FIGURE 7:**
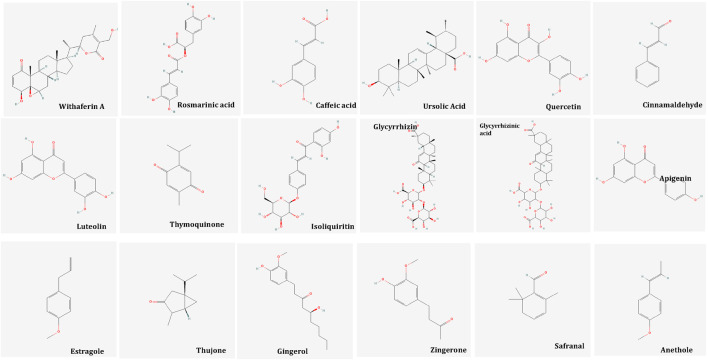
Bioactive Molecules in Unani Botanicals useful in Depression, Anxiety and Stress. Image from Pubchem.

Naturally occurring plant-based compounds, such as flavonoids and isoflavones, have demonstrated beneficial effects in alleviating menopausal symptoms, offering a safer alternative to hormone replacement therapy (HRT) with minimal adverse effects. These compounds, structurally similar to estrogen, exhibit estrogenic activity in human tissues. In addition to easing menopausal symptoms, flavonoids and isoflavones are believed to provide protection against chronic conditions such as breast cancer, osteoporosis, and cardiovascular diseases (Sultana et al., 2023). Numerous botanicals are useful for the alleviation of psychological symptoms in menopause. These are pharmacologically proven for innumerable properties such as antidepressant, sedative, anti-inflammatory, antioxidant, tranquillizing effect, analgesic, immune-modulator, CNS depressant and dopamine activities. Table 1 summarizes the findings of randomized controlled studies evaluating the use of botanical treatments for menopausal psychological symptoms. These studies focus on the efficacy of various plant-based interventions in alleviating symptoms such as anxiety, depression, and mood swings associated with menopause. *Withania Somnifera Dunal, Melissa officinalis* L., *Nigella sativa* L., *Tribulus terrestris* L., *Glycyrrhiza glabra* L., *Foeniculum Vulgare* Mill, *Zingiber officinale, Crocus sativus* extract*, Cinnamomum zeylanicum, Matricaria chamomilla, Lavandula officinalis L, Vitex agnus-castus, Pimpinella anisum L* and *Saliva officinalis* extract (Banu and Begum, 2012; Taavoni et al., 2017; Fatima and Sultana, 2017; Kashani et al., 2018; Mahdavian et al., 2019; Naseri et al., 2019; Dadfar and Bamdad, 2019; Rais et al., 2020; Ghaffari et al., 2020; Shirazi et al., 2021; Gopal et al., 2021; Lopresti and Smith, 2021; Azami et al., 2022; Taha and Dizaye, 2022; Bazrafshan et al., 2022; Begum and Sultana, 2023) (Table 1) These botanicals consist of various inorganic constituents and organic constituents including flavonoids, tannins, phenol, steroids, and protein) that have antioxidant, antidepressive, sedative, antianxiety and anti-inflammatory properties. Flavonoids possess anti-inflammatory effects primarily through their antioxidant properties and by modulating signal transduction pathways involved in the synthesis of proinflammatory cytokines. These compounds can neutralize reactive oxygen species (ROS), reducing oxidative stress, which in turn helps to lower inflammation. Additionally, flavonoids influence key signaling pathways, such as NF-κB and MAPK, that regulate the expression of cytokines, further contributing to their anti-inflammatory action (Naoi et al., 2019). The bioactive molecules are illustrated in Figure 7.

An antioxidant is a substance that helps prevent, slow down, or reverse oxidative damage to molecules by neutralizing ROS or other free radicals (Farràs et al., 2021). Flavonoids are well-known for their high antioxidant capacity, primarily due to their ability to scavenge free radicals and neutralize reactive oxygen species (ROS). This makes them effective in reducing oxidative stress and protecting cells from damage (Farràs et al., 2021). Flavonoids act as powerful antioxidants by directly scavenging reactive oxygen species (ROS) and reactive nitrogen species (RNS), while also reducing the production of free radicals and increasing the activity of ROS-removing enzymes. They neutralize free radicals such as hydroxyl, peroxyl, and superoxide radicals by producing hydrogen molecules or phenoxy radicals. The diol group in flavonoids can also prevent ROS generation by binding with transition metal ions like copper and ferric iron. Additionally, flavonoids upregulate enzymes like superoxide dismutase, catalase, glutathione peroxidase, and NAD(P)H: quinone oxidoreductase (NQO1). Flavonoids also exert anti-inflammatory effects by inhibiting the synthesis and release of proinflammatory molecules, such as nitric oxide, IL-6, TNF-α, and MCP-1. Antioxidants accomplish this by suppressing the activation of transcription factors such as NF-κB and AP-1, which are involved in the inflammation and oxidative stress response. Furthermore, flavonoids modulate second messenger pathways (e.g., cGMP, cAMP, calcium) to reduce the production of inflammatory mediators, such as PGs and ILs, and inhibit the activity of enzymes like COX and lipoxygenase (Naoi et al., 2019).

Flavonoids including quercetin and catechin can inhibit MAO-A, contributing to their antidepressant effects. Quercetin also activates upstream MAPK signaling, reducing induced apoptosis caused by oxidative stress and preventing Jun N-terminal kinase activation. Additionally, catechin increases BDNF serum levels and enhances the expression of TrkB and TrkA receptors, promoting neurogenesis and neuroprotection, further supporting its antidepressant activity (Naoi et al., 2019). Tannins may act as antioxidants by scavenging free radicals and preventing oxidative damage (Sofiane et al., 2015). Additionally, it is suggested that mitochondrial energy metabolism could play a role in the antidepressant mechanisms of action, potentially contributing to their therapeutic effects (Allen et al., 2018).

*W. somnifera:* Recent years have seen a notable increase in research highlighting the health benefits of *W. somnifera*, commonly known as Ashwagandha in Ayurveda or Asgandh in Unani. Studies have explored its neuroprotective, sedative, adaptogenic, and sleep-enhancing effects. Additionally, it has demonstrated anti-inflammatory, antistress, adaptogenic, neuroprotective, antidepressant, anxiolytic, cardioprotective, and anti-diabetic properties (Mikulska et al., 2023). A study used Prolanza™ and showed that taking one capsule daily of a sustained release capsule of Ashwagandha root extract (300 mg) for 90 days significantly improved overall psychological wellbeing. Stress levels were also reduced, and the treatment was well-tolerated and safe (Gopukumar et al., 2021). One study investigated the use of Ashwagandha extract alongside SSRIs in patients diagnosed with GAD. Participants took one capsule of Ashwagandha extract daily for 6 weeks. The results indicated that Ashwagandha extract could potentially complement SSRI therapy for GAD patients. Notably, supplementation with Ashwagandha significantly reduced scores on the Hamilton Anxiety Rating Scale (HAM-A) and, to a lesser extent, on the Depression, Anxiety, and Stress Scale (DASS-21) (Mikulska et al., 2023; Pratte et al., 2014). Ashwagandha’s anxiolytic effects may stem from various mechanisms. It may reduce the activity of the hypothalamic–pituitary–adrenal (HPA) axis, which in response to stress increases cortisol and DHEA levels. DHEA, a hormone that declines with age, plays a role in managing psycho-physical and psychosexual issues during menopause and andropause. Elevated DHEA is linked to chronic stress and overactivity of the HPA axis, as well as behaviors like smoking and alcohol consumption. Ashwagandha’s anti-inflammatory and antioxidant potential also contribute to its ability to reduce stress, depression, and anxiety by targeting these processes simultaneously (Mikulska et al., 2023). Sleep-deprived rats showed increased expression of pro-inflammatory markers, while Ashwagandha treatment inhibited stress-induced apoptosis and enhanced the expression of AP-1, NF-κB, Bcl-Xl, and cytochrome C (Kaur et al., 2017). Significant improvements in cognitive function were observed, likely due to the inhibition of amyloid β-42 and a decrease in pro-inflammatory cytokines, including TNF-α, IL-1β, IL-6, MCP-1, as well as reductions in nitric oxide and lipid peroxidation.

Additionally, the activity of β- and γ-secretase enzymes, which contribute to the formation of neurotoxic β-amyloid aggregates, was reduced. Withaferin A, an active compound in Ashwagandha, shows promise for Alzheimer’s treatment by decreasing β-amyloid aggregation and inhibiting τ protein accumulation. It also inhibits oxidative and pro-inflammatory factors while regulating heat shock proteins (HSPs), which are activated by cellular stress. However, further studies are needed to evaluate the safety and confirm the neuroprotective effects of withaferin A in Alzheimer’s treatment (Mikulska et al., 2023). *W. somnifera* has been linked to adverse effects when combined with certain antidepressants, mostly SSRIs like escitalopram, paroxetine, and sertraline. These effects possibly result from its potential to inhibit or alter cytochrome P450 enzymes (CYP3A4, CYP2D6), leading to increased drug levels and overlapping side effects including gastrointestinal distress and myalgia. While *in vitro* studies suggest possible herb–drug interactions, findings are inconsistent, and further clinical research is needed to confirm these effects (Siwek et al., 2023).

*M. officinalis* L.: It is an aromatic perennial medicinal herb, commonly known as honey balm, lemon balm, or balm mint, widely used in traditional medicine across the globe. It is from the mint family (Lamiaceae). *M. officinalis* has many phytoactive molecules including volatile compounds such as citronellal, neral, geranial, and geraniol, triterpenes like oleanolic and ursolic acid, phenolic acids including rosmarinic acid, and chlorogenic acid, as well as flavonoids such as quercetin, and luteolin (Petrisor et al., 2022)*.* Various pharmacological activities include antioxidant, anxiolytic, antidepressant, neuroprotective, GABA-T inhibitor, analgesic and so on (Noorul Basar and Zaman, 2013). It is known for its anxiolytic effects, and has been shown to improve sleep quality in humans, particularly in cases of sleep disturbances during menopause. It has also been tested on animal models for its ability to reduce anxiety and act as an antioxidant. The mechanism of action is believed to involve the elevation of gamma-aminobutyric acid (GABA) levels in the brain, as GABAergic neurotransmission has been associated with reduced anxiety (Shirazi et al., 2021). A study found that acute administration of 600 mg of *M. officinalis* extract increased calmness and reduced alertness, suggesting the involvement of the GABAergic system in its anxiolytic effects. A recent survey of ten anxiolytic botanicals identified *M. officinalis* extract as the most effective inhibitor of *in vitro* GABA-T activity in rat brains. Similarly, another study reported that *M. officinalis* inhibits GABA-T, attributing this effect to rosmarinic acid, a major component of lemon balm. Our study confirmed that *M. officinalis* inhibits GABA-T activity; however, this mechanism alone may not be sufficient to significantly counteract the stress-induced reduction of GABA levels (Scaglione and Zangara, 2017). However, the full extent of its therapeutic effects and the amounts of various constituents in the extract remain unknown (Shirazi et al., 2021).

*N. sativa:* Black seed possesses notable antioxidant, immunomodulatory, anti-inflammatory, and anticancer properties. The total antioxidant activity (TAA) is attributed to the residual activity in cell supernatants and the presence of thymoquinone in its extracted oil. Thymoquinone (TQ) has been shown to significantly reduce levels of proinflammatory cytokines, such as IL-6, which is implicated in various psychological confditions, including depression and anxiety. By modulating inflammatory pathways and oxidative stress, *N. sativa* may offer therapeutic potential for managing psychological symptoms, particularly those associated with neuroinflammation (Bordoni et al., 2019; Hannan, Abdul, Ataur Rahman et al., 2021). *N. sativa* has role against neuroinflammation. The activation of NF-κB transcription factors through DNA binding plays a pivotal role in controlling the expression of pro-inflammatory cytokines. Once activated, NF-κB drives the transcription of genes responsible for producing pro-inflammatory cytokines, chemokines, and enzymes. Hence, pharmacological approaches aimed at modulating microglial activation offer promising therapeutic prospects for managing neuronal disorders associated with inflammation.

In BV-2 microglial cells activated by LPS/IFNγ or H_2_O_2_, thymoquinone (TQ) treatment (12.5 µM for 24 h) reduced oxidative stress and inflammation by increasing levels of GSH, SOD, and CAT, while decreasing lipid hydroperoxides, cytokines and chemokines. Additionally, TQ inhibited LPS-induced neuroinflammation in BV-2 microglia by activating LK1, AMPK, and SIRT1. Its anti-inflammatory effects, particularly TQ’s role in mitigating neuroinflammation, suggest its potential as a therapeutic agent for treating inflammation-mediated neurological disorders (Hannan, Abdul, Ataur Rahman et al., 2021).

*T. terrestris* L: *T. terrestris* (Zygophyllaceae family), is widely cultivated in the Mediterranean, subtropical regions, and countries like India. Known by local names such as goat head and hard thorns, *T. terrestris* has long been a key component of traditional health systems. Pharmacological studies have validated its traditional uses, demonstrating its effectiveness in treating inflammation, skin dryness and itchiness, heart and vascular issues, carcinomas, microbial infections, oxidative stress, hormonal imbalances, and muscle repair. These therapeutic effects are attributed to its active compounds, including saponins, flavonoids, and alkaloids. It has pharmacological properties including antioxidant, anti-inflammatory, anxiolytic, diuretic, phytoestrogenic and other therapeutic properties (Abbas et al., 2022). The anti-inflammatory potential observed in the *T. terrestris* contain total phenolic and flavonoid contents. The presence of phytochemicals such as stilbenes, anthocyanins, tannins, alkaloids, and steroids in plants has been reported to contribute to their anti-inflammatory properties (Huang et al., 2017). It is well-established that abnormalities in neuroendocrine function play a significant role in the onset of depression. The HPA axis is a critical neuroendocrine system, and its hyperactivity is regarded as a key neurobiological feature of major depression. *T. terrestris* has been reported to inhibit the CYP3A4 enzyme. This inhibition probably elevates blood levels of certain antidepressants metabolized by CYP3A4, possibly intensifying their side effects. A limited case reports have linked its concomitant use with citalopram, escitalopram, and trazodone to adverse events such as pruritus, galactorrhea, and psoriasis relapse. Although formal clinical studies on such herb–drug interactions are lacking, caution is advised when combining *T. terrestris* with CYP3A4-metabolized medications (Siwek et al., 2023).

Corticotropin-releasing factor (CRF) regulates the HPA axis and is linked to depressive symptoms. Elevated cortisol levels often normalize with symptom improvement after antidepressant treatment. Therefore, the normalization of HPA axis activity serves as a key therapeutic target and an indicator of depression recovery. A study reported that TTS at doses of 0.75 and 2.25 g/kg significantly reduced CMS-induced increases in serum CRF and CORT levels, indicating its ability to normalize HPA axis hyperactivity. These findings suggest that TTS may exert antidepressant effects by modulating the HPA axis. Further preclinical and clinical studies are needed to confirm its therapeutic potential (Wang et al., 2013).

The ethanolic extract of *T. terrestris* (TT) has been shown to inhibit the expression of COX-2 and iNOS in lipopolysaccharide-stimulated RAW264.7 cells. It also suppresses the production of proinflammatory cytokines, including TNF-α and IL-4, in a macrophage cell line. The ethanolic extract of TT may inhibit inflammatory mediators and cytokines, offering potential therapeutic benefits for inflammatory conditions (Chhatre et al., 2014). Serum superoxide dismutase (SOD) and malondialdehyde (MDA) activity were restored following administration of high and medium doses of TT extract. In addition, SOD and GSH-Px activity in the brain tissue of treated aging mice showed significant increases compared to non-treated aging mice. Concurrently, catalase (CAT) activity and MDA levels in brain tissue were significantly reduced in the treated groups. These findings suggest that TT extract may enhance antioxidant defense mechanisms and mitigate oxidative stress in aging mice. The results provide valuable insights for the clinical application and further investigation of *T. terrestris* (Tian Mei et al., 2021).

*G. glabra L.:* Licorice, cultivated extensively in the Asia, Middle East, and Europe, is rich in bioactive compounds like glycyrrhizic acid, glycyrrhizin, and isoliquiritin, offering diverse therapeutic benefits. It has anti-atherogenic, anticancer, antidiabetic, antispasmodic, anti-inflammatory, and anti-asthmatic effects.

Licorice, known for its versatility, has been used to manage cognitive impairment, dementia, and Alzheimer’s disease. Its roots, extracts, and active compounds, such as flavonoids, and glycyrrhizic acid, demonstrate potential benefits for respiratory health, immune support, anti-cancer activity, inflammation reduction, and gastrointestinal and liver protection. These attributes make licorice a vital herb and a central focus of contemporary herbal medicine research. In traditional medicine, it is beneficial as anti-inflammatory effects. Its active compounds, including glycyrrhizin, isoliquiritigenin, and flavonoids, modulate various inflammatory pathways. Glycyrrhizin suppresses the expression of inflammatory markers like iNOS, COX-2, TNF-α, IL-6, and IL-1β, attenuating inflammation and oxidative stress. Isoliquiritigenin, a flavonoid, alleviates oxidative stress by activating the Nrf2/HO-1 pathway and reduces inflammation in conditions like acute pancreatitis and kidney damage. Licorice also inhibits matrix metalloproteinases (MMPs), NF-κB, and other pro-inflammatory molecules, demonstrating its broad anti-inflammatory potential. Furthermore, licorice extracts have been shown to reduce oxidative stress in bronchial asthma and suppress inflammation in rheumatoid arthritis (RA) through TLR4/NF-κB/NLRP3 signaling. These findings highlight licorice as a promising natural remedy for treating various inflammatory diseases with minimal adverse effects. Quercetin inhibits inflammatory pathways by competing for ATP binding sites, thereby preventing the activity of various protein and lipid kinases. This mechanism highlights its potential as an anti-inflammatory agent, capable of modulating key signaling processes involved in inflammation (Wahab et al., 2021; Liu et al., 2018). Licorice, rich in triterpenoids and flavonoids, contains key active compounds such as licorice total flavonoids (LF) and liquiritin, demonstrated significant antidepressant effects.

These compounds achieve their therapeutic effects via multiple mechanisms. LF and liquiritin improve depressive behaviors by regulating the endocrine and hypothalamic-pituitary-adrenal (HPA) axis function, which plays a critical role in stress responses. They also modulate the BDNF/TrkB signaling pathway, which is involved in neurogenesis and synaptic plasticity. Additionally, these compounds enhance the levels of monoamine neurotransmitters, protect nerve cells, reduce inflammation, prevent apoptosis, and counteract oxidative stress. These mechanisms collectively contribute to the antidepressant potential of licorice and its bioactive constituents in treating major depressive disorder (MDD) (Wang et al., 2023).

*Foeniculum vulgare:* Fennel is a widely recognized aromatic medicinal plant and culinary herb. It has been shown to alleviate gastrointestinal dysfunction and dysmenorrhea, relax smooth muscles, boost breastmilk production, and enhance age-related memory deficits. It contains estragole, α-pinene, β-fenchol, limonene, α-terpinene, α-thujone, and β-myrcene (Ogbonna et al., 2024). Additionally, fennel exhibits anti-inflammatory, antiandrogenic, and anxiolytic properties, effectively reducing anxiety. Clinical trials have demonstrated its potential in managing menopausal symptoms, including hot flushes, stress, sleep disorders, and vaginal atrophy, yielding positive outcomes (Lee et al., 2021). A study investigated the anti-stress and memory-enhancing properties of *F. vulgare* extract in lab rats. Anti-stress activity was evaluated by measuring urinary levels of VMA and ascorbic acid. Daily administration of the extract 1 hour before stress induction significantly reduced stress-induced urinary VMA levels in a dose-dependent manner, suggesting its potential in managing stress and related disorders (Fattah, 2017). A systematic review reported the effectiveness and safety of fennel for menopausal health. A search across 14 databases identified seven RCTs meeting the inclusion criteria. While two RCTs demonstrated fennel’s significant benefit over placebo in improving menopausal symptoms, other studies showed no clear advantages for sexual function, quality of life, or psychological health. Although fennel appears promising for alleviating menopausal symptoms, its broader impact on quality of life and psychological wellbeing remains uncertain (Lee et al., 2021). Fennel exhibits significant anti-inflammatory effects by reducing calcium influx recovery time and suppressing the phosphorylation of MAPKs, including JNK, ERK, and p38, thereby inhibiting degranulation and respiratory bursts in human neutrophils. H89-mediated inhibition of PKA partially restores superoxide anion generation, suggesting the involvement of the cAMP/PKA pathway in fennel’s anti-inflammatory effects. Furthermore, fennel’s estragole suppresses Nrf-2 and NF-κB pathways *in vitro* and, at a dose of 30 mg/kg *in vivo*, reduces paw edema and leukocyte migration in peritoneal fluid (Korinek et al., 2021).

*Z. officinale* Roscoe: *Z. officinale* Roscoe, a member of the Zingiberaceae family, has long been used both as a spice and an herbal remedy for various ailments. The root of ginger contains bioactive compounds, including phenolic compounds like gingerols, shogaols, and paradols, which contribute to its diverse biological activities. These activities include anti-inflammatory, antidepressant, antioxidant, anxiolytic, anticancer properties and so on. Recent research highlights ginger’s potential in preventing and managing a range of conditions, include neurodegenerative, and cardiovascular conditions disorder. Ginger’s broad therapeutic applications underscore its importance as a functional food and medicinal plant (Mao et al., 2019). Studies have shown that ginger effectively protects against oxidative stress. Its antioxidant mechanisms have been explored in various cell models, including human chondrocyte cells, where it reduces oxidative stress induced by IL-1β by enhancing antioxidant enzyme expression and decreasing reactive oxygen species (ROS) production and lipid peroxidation. The antioxidant activity of ginger and its bioactive compounds, like 6-shogaol, is thought to be mediated by the nuclear factor Nrf2 signaling pathway, boosting the body’s defense against oxidative damage (Romero et al., 2018). Recent investigations indicate that ginger improves memory and has anti-neuroinflammatory effects, which may aid in managing and preventing neurodegenerative diseases.

A study using a lipopolysaccharide (LPS)-activated BV2 microglia culture model revealed that 10-gingerol, a major bioactive compound in ginger, was responsible for its strong anti-neuroinflammatory effects. It inhibited the activation of NF-κB, leading to a reduction in proinflammatory cytokines such as NO, IL-1β, IL-6, and TNF-α. Additionally, in mice with scopolamine-induced memory deficits, ginger extract improved cognitive function, as assessed by the novel object recognition test. Further studies in mouse hippocampi and rat C6 glioma cells demonstrated that ginger extract promoted synapse formation in the brain by activating extracellular signal-regulated kinase (ERK) signaling, which is induced by nerve growth factor (NGF) and cyclic AMP response element-binding protein (CREB). These findings suggest that ginger may offer potential therapeutic benefits for cognitive impairments and neuroinflammation (Mao et al., 2019; Lim et al., 2014). A study using LPS-activated BV2 microglia showed that 10-gingerol, a key compound in ginger, exerted strong anti-neuroinflammatory effects by inhibiting NF-κB activation, leading to reduced proinflammatory cytokines (NO, IL-6, IL-1β, TNF-α). These findings suggest that ginger may provide therapeutic benefits for cognitive impairments and neuroinflammation. The ginger rapidly increases hippocampal BDNF expression. Aphrodite herbal, rich in compounds like gingerol and zingerone, exhibits anxiolytic effects by interacting with GABA receptors in the brain, enhancing GABAergic activity. GABA, an inhibitory neurotransmitter, helps regulate anxiety and stress by reducing neuronal excitability. Similarly, saffron, containing compounds like crocin and safranal, affects serotonin levels in the brain. Since serotonin plays a key role in mood regulation, its imbalance is linked to anxiety disorders (Shahmoradi et al., 2023).

*C. sativus*: *C. sativus* exhibits antioxidant, cardioprotective, neuroprotective, hypolipidemic, antidepressant, antihyperglycemic effects, and protection against retinal damage. Its antioxidant and antimicrobial activities also make it an effective natural food preservative. In India, it is applied against depression and similar mental disorders (Butnariu et al., 2022). Research indicates that saffron possesses notable antioxidant properties. Among its phytoconstituents, crocetin exhibits stronger antioxidant activity compared to safranal and demonstrates a potency comparable to that of Trolox and butylated hydroxytoluene (BHT) (Butnariu et al., 2022). The findings of this study align with prior clinical trials that highlight saffron’s antidepressant properties (Butnariu et al., 2022; Kashani et al., 2017; Agha-Hosseini et al., 2008). These effects are driven by its serotonergic, anti-inflammatory, neuroendocrine, antioxidant, and neuroprotective actions (Lopresti and Drummond, 2014). Studies using rat models suggest that saffron’s antidepressant effects may result from increased levels of BDNF, VGF neuropeptide, CREB, and p-CREB in the hippocampus (Ghasemi et al., 2015). Saffron and its components exhibit significant antidepressant and anxiolytic effects. Crocin reduces immobility time and increases climbing behavior in rats, while extracts from saffron petals and stigmas, along with compounds like safranal and kaempferol, also demonstrate antidepressant activity in rodents. These effects are likely mediated through mechanisms similar to selective serotonin reuptake inhibitors, enhancing serotonin activity (Butnariu et al., 2022). Saffron’s bioactive compounds are believed to boost serotonin levels by inhibiting its reuptake, extending its presence in the synaptic spaces. This increase in serotonin transmission is thought to contribute to saffron’s anxiolytic effects, promoting relaxation and reducing anxiety symptoms (Shahmoradi et al., 2023).

*C. zeylanicum* Blume: *C. zeylanicum* Blume, or true cinnamon, is widely recognized for its therapeutic properties and distinct spicy flavor, due to its exceptional chemical composition, and botanical member of the Lauraceae family. It is believed to have originated in the central hills of Sri Lanka (Madhushika et al., 2024). *C. zeylanicum* has been linked to a range of potential medicinal effects, including anti-mutagenic, antioxidant, anti-inflammatory, anticancer, anti-tyrosinase, antidepressant, and alleviation of neurological disorders (Balijepalli et al., 2017; Yulug and Cankaya, 2019; Abdollahi et al., 2020; Aryanezhad et al., 2021). Cinnamaldehyde inhibits NO production, a key factor in inflammatory diseases, and reduces COX-2-mediated prostaglandin E2 biosynthesis. A 70% ethanolic extract of cinnamon has shown effectiveness in reducing acute inflammation in mice. Furthermore, an herbal ophthalmic preparation, Ophtha Care, containing 0.5% cinnamon, demonstrated anti-inflammatory properties for ocular inflammation in rabbits (Das et al., 2016). The antidepressant effect is attributed to terpene and monoterpenoid compounds such as beta-pinene, beta-thujone, limonene, and linalool. These compounds are also the primary constituents of *C. zeylanicum*, which has demonstrated antidepressant properties through the inhibition of monoamine oxidase A and B. Additionally, *C. zeylanicum*, due to its beta-pinene content, can enhance animal activity by increasing dopamine levels and reducing monoamine oxidase activity. One of the most notable neuroanatomical changes in depression is the reduction in the size of the hippocampus in affected individuals. It has been shown that certain antidepressants contribute significantly to cell regeneration by stimulating the expression of neurotrophic factors. The most important of these growth factors is BDNF, which is increased by antidepressants in the hippocampus (Aryanezhad et al., 2021). Cinnamon contains compounds like cinnamaldehyde and eugenol, which have notable effects on CNS disorders. Eugenol has been shown to enhance neurotrophic factor expression in the hippocampus, promoting brain cell regeneration. It also increases the expression of metallothionein-3, a neuroprotective protein. Therefore, the antidepressant effects of cinnamon may be attributed to eugenol. Additionally, cinnamaldehyde contributes to pain relief and stimulates the central nervous system (Das et al., 2016). Studies have shown that cinnamon compounds exert significant anti-Alzheimer effects by improving insulin signaling and cognitive function. In experimental models of Alzheimer’s and diabetes, cinnamon demonstrated pro-cognitive effects by reducing oxidative stress and enhancing acetylcholinesterase activity. It also suppressed β-secretase enzyme activity, confirming its anti-amyloid properties. Additionally, cinnamon regulated glucose levels and exhibited insulinomimetic effects through signaling proteins and glucose transporter expression (Yulug and Cankaya, 2019). Cinnamon could serve as a potential therapeutic agent for neurodegenerative disorders, commonly associated with increased inflammation and oxidative stress, because of its antioxidant, anti-inflammatory, and neuroprotective properties (Balijepalli et al., 2017).

*M. chamomilla*: *M. chamomilla* tea exhibits a range of beneficial effects on psychological symptoms, attributed to its anti-inflammatory, anxiolytic, antioxidant, analgesic, and antidepressant properties. The flavonoid apigenin, a major compound in chamomile, modulates neurotransmitter activity by reducing the effects of stress-related hormones and inhibiting excitatory neurotransmitters, thereby attenuating sympathetic nervous system hyperactivity. Apigenin also influences dopaminergic and serotonergic pathways, contributing to the reduction of depressive symptoms. Furthermore, chamomile exerts analgesic effects by inhibiting cyclooxygenase (COX) enzymes and suppressing immune-mediated inflammation. Its anxiolytic and stress-relieving properties are further supported by compounds such as apigenin, luteolin, glycine, and other flavonoids, which act as central nervous system relaxants. Overall, chamomile is considered a safe and effective herbal intervention for managing stress, anxiety, and associated psychological symptoms (Khalesi et al., 2019).

*L. officinalis* L: It is a member of the Lamiaceae family and belongs to the genus Lavandula, which comprises approximately 28 species. It has been used for centuries to treat various conditions, nerve pain, including scalds, rheumatism, diarrhea, cold, cough, and respiratory issues like asthma. It is also used to relieve fatigue, support recovery, and alleviate depression, stress, anxiety, and insomnia. Known for its strong antibacterial, anti-inflammatory, anxiolytic, sedative, antidepressant, analgesic, anticonvulsive and neuroprotective potenitial, it is particularly effective in reducing pain and inflammation, possibly through COX enzyme inhibition (Diass et al., 2023; Afsahul Kalam, et al., 2021; Koulivand et al., 2013). Lavender essential oil (LEO) has been compared to medications like lorazepam, paroxetine, and diazepam in studies on anxiety, both in humans and animals. In a study on GAD, gelatin capsules containing LEO (Silexan) were found to be more effective than lorazepam in reducing anxiety symptoms after 6 weeks. Unlike benzodiazepines, LEO poses no risk of abuse or withdrawal, making it a safer alternative for managing anxiety (Naghdi et al., 2024). This systematic review and meta-analysis included 17 studies and evaluated the antidepressant effects of lavender. The results showed significant reductions in depression scores with lavender compared to controls (SMD = −0.66, p < 0.001) (Firoozeei et al., 2021). The anti-anxiety effects of lavender are primarily attributed to its volatile compound, linalool (Naghdi et al., 2024). Linalool, the primary constituent of lavender, acts as a sedative by inhibiting glutamate binding. Additionally, pennine, a key component of both lemon and lavender, enhances sedative effects when combined with gamma-aminobutyric acid (GABA) (Herz, 2009). Lavender oil is believed to influence GABAergic neurotransmission, especially at GABAA receptors, by enhancing the inhibitory activity of the nervous system. Furthermore, the cholinergic system is thought to play a role in lavender’s anxiolytic, antidepressant, and anticonvulsant effects (Koulivand et al., 2013). Lavender may impact depression by blocking glutamate NMDA receptors, inhibiting serotonin transporter proteins, reducing binding to 5HT1A receptors, and suppressing voltage-gated calcium channels (VGCCs). These effects suggest lavender as an effective supplemental treatment for depression, not only in those diagnosed with depression but also in patients with other conditions and concurrent depressive symptoms (Firoozeei et al., 2021).

*Vitex agnus-castus*: Chaste tree is a shrub or small tree, growing 3–6 m tall. It is classified in the Lamiaceae family, though it was once part of the Verbenaceae family. Its extract contains 10 iridoids, 20 flavonoids, three diterpene alkaloids, 11 phenolic acids, and 12 terpenes, exhibit pharmacological activities, including estrogenic, antioxidant, dopaminergic, opioid, immunomodulatory, and antineoplastic effects as confirmed by preclinical studies (Adamov et al., 2022). Jarry et al. found that the flavonoids apigenin, and vitexin exhibited estrogenic effects. These compounds specifically showed affinity for the β-estrogen receptors (Jarry et al., 2006). Arokiyaraj et al. found that the aucubin iridoid agnuside from chaste tree extract significantly inhibited iron II-mediated free radical oxidation of deoxyribose (Arokiyaraj et al., 2012). Plants containing phytoestrogens, which are estrogen-like compounds, play a key role among herbs recommended for treating menopausal symptoms, which are structurally similar to estrogens and exert dopaminergic effects by binding to dopamine D2 receptors. Its phytoestrogens have a higher affinity for beta estrogen receptors, which are primarily found in the ovaries, uterus, brain, and bladder, helping to restore hormonal balance in women. Studies have also reported a positive correlation between menopausal symptoms and anxiety, depression, and mental health scores (Arokiyaraj et al., 2012).

*P. anisum* L: *P. anisum* commonly known as aniseed, is an aromatic medicinal plant from the Apiaceae family, primarily used to treat female disorders. A review of 56 studies, highlighted the effectiveness of aniseed in managing conditions such as postpartum pain, menstrual pain, PCOS, lactation issues, and depression. The fruits of this plant are rich in essential oil, containing 1.5%–6% oil, primarily composed of trans-anethole (85%–90%), along with smaller amounts of estragole, pinenesand limonene. The key therapeutic properties of aniseed include its analgesic, anti-inflammatory, estrogenic, analgesic, anticonvulsant, antidepressant, anxiolytic and antispasmodic effects, along with its ability to inhibit prolactin secretion. The essential oil and ethanol extract of aniseed is primarily responsible for its therapeutic efficacy (Shojaii and Fard, 2012; Sun et al., 2019). However, most studies focus on its combination with other plant materials or specific female disorders (Mahboubi and Mahboubi, 2021). Phytoestrogens, like aniseed, can help balance estrogen levels in the body and significantly reduce the severity and frequency of hot flashes. Anethole is believed to exert estrogen-like effects in the body, which may contribute to its effectiveness in managing symptoms associated with hormonal imbalances, such as those experienced during menopause. Its potential as a phytoestrogen helps balance estrogen levels, alleviating issues like hot flashes, menstrual discomfort, and other estrogen-related conditions (Helal et al., 2019). Depression frequently occurs before hot flashes due to hormonal changes during menopause. Both ethanol and aqueous extracts of aniseed have demonstrated antidepressant effects in animal and human studies. This is likely due to the higher solubility of anethole in ethanol, which contributes to the antidepressant properties of the ethanol extract (Shahamat et al., 2014). The total extract of *P. anisum* (PATE) at doses of 100 and 200 mg/kg demonstrated significant anxiolytic and antidepressant effects in Swiss albino mice, as shown by reduced immobility and downtime in tests. Memory tests (NORT and MWMT) indicated no adverse impact on cognitive function. Phytochemical analysis via HPLC identified bioactive compounds, including gallic acid, catechin, chlorogenic acid, caffeic acid, and quercetin. The study supports the traditional use of PATE as a potential phytomedicine for anxiety and depression and encourages further investigation into its mechanisms of action (Es-safi et al., 2021). Additionally, antioxidant compounds in aniseed may contribute to its antidepressant activity by inhibiting the reuptake of serotonin and monoamine oxidase, thus increasing serotonin levels in the synaptic clefts and enhancing receptor sensitivity. The mechanism of action for aniseed ethanol extract is similar to that of fluoxetine, making it a clinically valuable option for managing depression during menopause (Mahboubi and Mahboubi, 2021). A positive correlation was observed between the antioxidant activity and the flavonoid content in the extracts (Shojaii and Fard, 2012).

**FIGURE 8:**
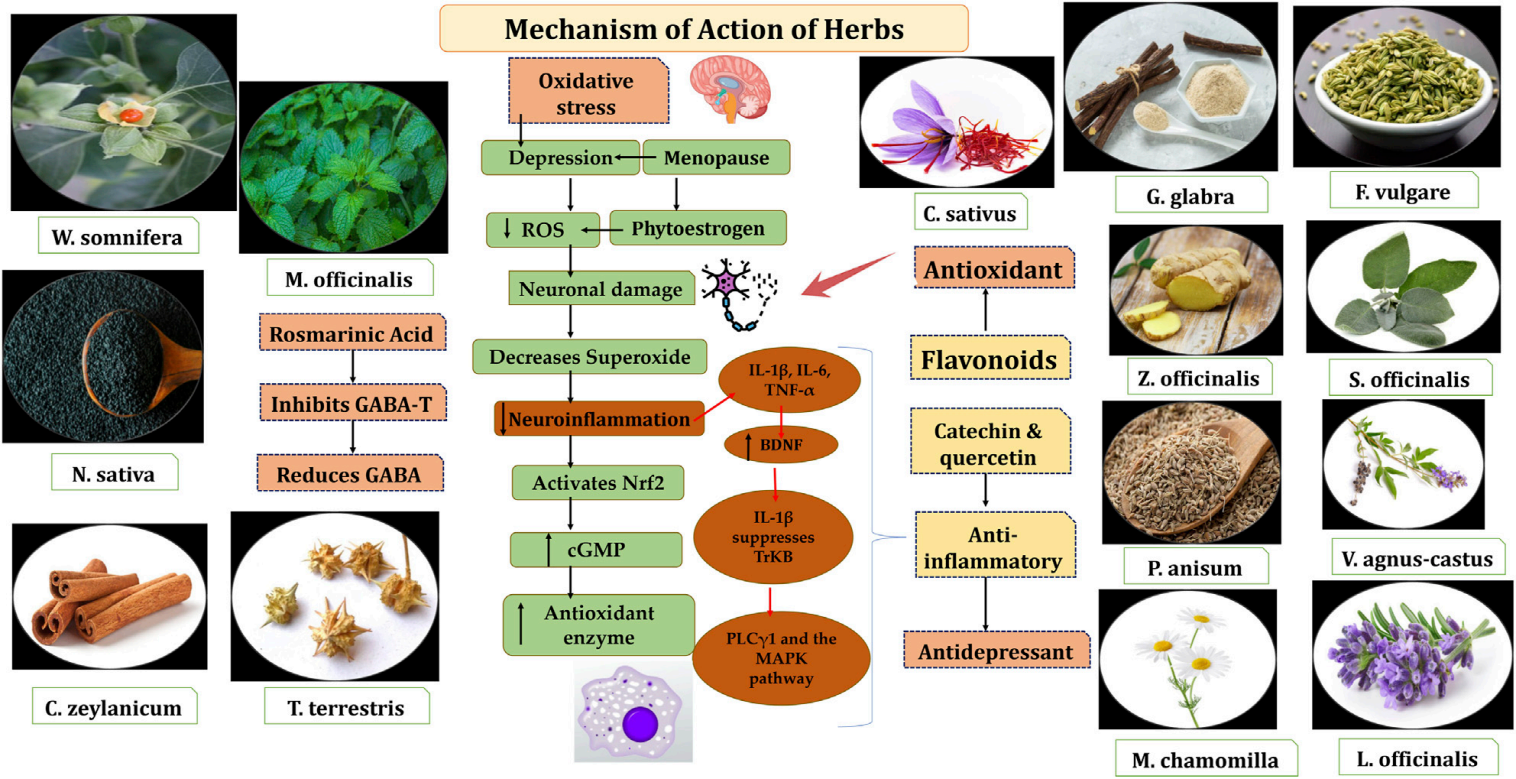
Depiction of mechanism of action of herbs as antioxidant and anti-inflammatory in depression. Source Plant image from freepik.com.

*Saliva officinalis:* Sage (Lamiaceae family) native to the Middle East and Mediterranean, has been traditionally used to treat seizures, rheumatism, and hyperglycemia. Recent pharmacological studies include anti-inflammatory, antinociceptive, antioxidant, and antidementiaeffects (Ghorbani and Esmaeilizadeh, 2017; Tayebi et al., 2024). *S. officinalis* contains flavonoids like rosmarinic acid, ellagic acid, and quercetin, volatile compounds such as borneol, camphor, cineole, and thujone. Rosmarinic acid is the most abundant flavonoids, while arabinose, galactose, and glucose are the primary carbohydrates. Linalool is the most prevalent compound in the stem, α-pinene and cineole are highest in the flowers, and the leaves contain bornyl acetate, camphene, and thujone (Ghorbani and Esmaeilizadeh, 2017). Studies have identified various active compounds in Salvia species improve cognitive function and protect against neurodegenerative diseases. The active constituents of Salvia plants and their effects on cognitive abilities such as memory, attention, and learning. Salvia constituents can influence multiple biological mechanisms related to cognition, such as effects on amyloid-β, neurotrophins, inflammation, cholinergic activity, oxidative stress, and behaviors associated with anxiety and depression (Lopresti, 2017). *S. officinalis* exhibits potent antioxidant properties. It protects against DNA damage induced by dimethoxy naphthoquinone and hydrogen peroxide by boosting glutathione peroxidase activity. Key antioxidant compounds include carnosol, rosmarinic acid, and carnosic acid, followed by rosmanol and caffeic acid (Horváthová et al., 2016). Flavonoids and terpenes in *S. officinalis* likely contribute to its anti-inflammatory and antinociceptive effects. Studies show that flavonoids extracted from *S. officinalis* reduce inflammation in the mouse carrageenan model and induce a dose-dependent analgesic effect. Additionally, rosmarinic acid has been shown to inhibit epidermal inflammation when applied topically (Mansourabadi et al., 2015). Phenolic diterpenes, carnosol and carnosic acid, present in *S. officinalis* significantly inhibited the gene expression of iNOS, cytokines/interleukins (IL-1α, IL-6), and chemokines such as CCL5/RANTES and CXCL10/IP-10 (Schwager et al., 2016). *S. officinalis* reduced AChE activity in mice with more significant effects on butyrylcholinesterase (Scholey et al., 2008). The mechanism of actions is depicted in Figure 8.

The mechanisms behind the phytoestrogen effects in menopausal symptoms are not fully understood, but phytoestrogens may exert their effects through mechanisms independent of estrogen receptors. Various studies have shown that phytoestrogens can bind to estrogen receptors and exhibit noteworthy estrogen-like effects (Parhizkar et al., 2011). The mechanism of action of anise is similar to selective estrogen receptor modulators, exhibiting both agonistic and antagonistic effects on estrogen receptors. Anethole, the active estrogenic compound in anise, contributes to its phytoestrogenic properties (Farahmand et al., 2021). Similarly, *N. sativa* may influence estrogen receptors both directly and indirectly, potentially altering estrogen levels. Its estrogenic activity is likely attributed unsaturated fatty acids, which are known to have estrogenic effects (Parhizkar et al., 2011; Allen et al., 2018).

**FIGURE 9:**
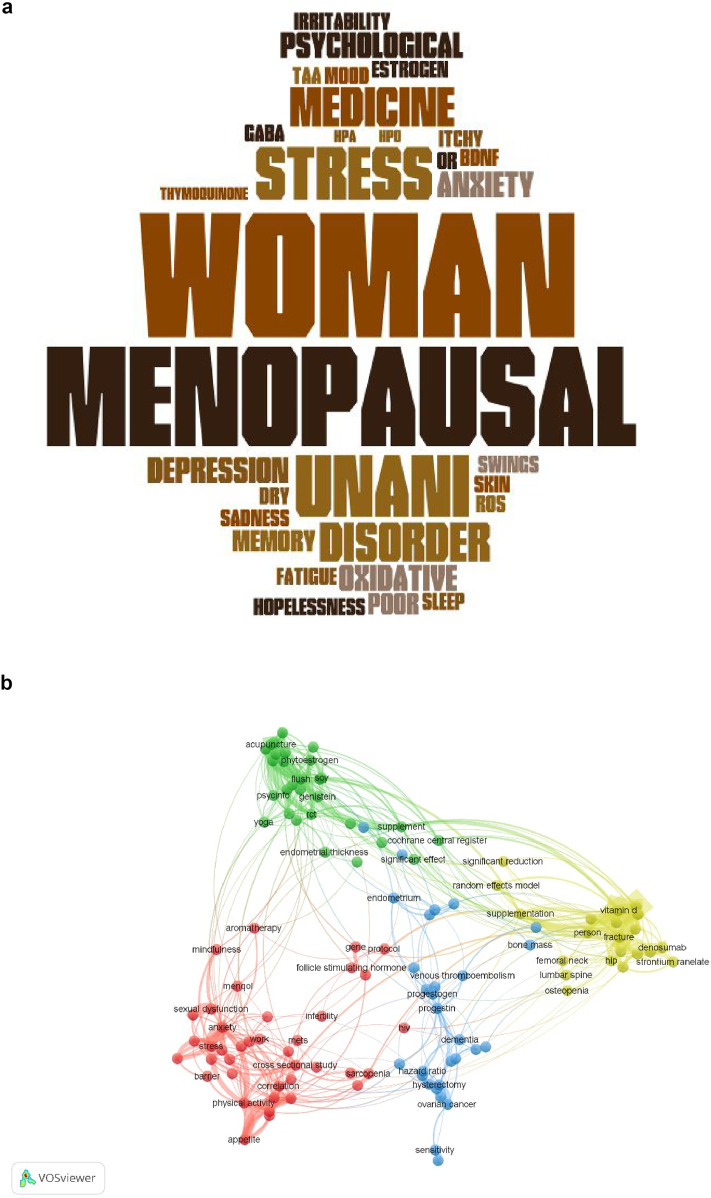
Closest terms based on **(a)** present study using word cloud, and **(b)** previous study using network visualization.

This review addressed research questions on oxidative stress, inflammation, antioxidants, and anti-inflammatory botanicals, and explored the pharmacological activities and mechanisms of botanicals in menopausal symptoms. This review highlights high quality of 16 RCTs on menopausal psychological symptoms and focusing on pathophysiology of menopausal psychological symptoms. We reviewed 16 studies with 1112 participants (mean age: 69.5 ± 21.88) using PRISMA methods. Additionally, we used network visualization to analyze MeSH terms and cluster related data (Sheikh et al., 2021) (Table 2; Figure 9). This technique is useful for identifying precise MeSH keywords related to specific diseases and offers a new approach to analyzing previously published data. Manually selecting the closest terms from large databases can be difficult, but these tools provide an efficient way to visualize and organize the dataset using software. They allow for easy clustering of related articles. Relevant studies were obtained from PubMed, Science Direct, and PROSPERO databases to further explore inflammation, oxidative stress, and the role of Unani botanicals.

**TABLE 2:** Closest terms identified through previous studies.

S. No.	Research questions	Closest terms
1	What is the pathophysiology of psychological Symptoms (Depression, Anxiety and Stress) in Menopause?	Anxiety, depression, stress, patient, pain, placebo, Perimenopausal, postmenopasual symptoms, menopausal syndrome, relationship, severity, symptom, climacteric, behavioural, woman, HPA, HPO, hormonal changes, menopausal transition, FSH, LH, Inhibin, progesterone, estrogen
2	What is the role of neuroinflammation and oxidative stress in depression associated with menopause?	Anxiety, biomarkers, inflammation, antioxidants, depression, experimental trials, clinical trials, neuro-inflammation, menopausal syndrome, BDNF, Interleukin, roots, superoxide, symptom, woman, BDR, brain, symptom, PMS, protein, ROS, woman, MDD, IL-8, TNF-α, MAPK, RNS, Nrf2, estrogen, cGMP
3	What is the mechanisms of action of bioactive molecules of Unani botanicals in psychological symptoms in menopause?	Anxiety, experimental trials, clinical trials, depression, inflammation, neuro-inflammation, patients, menopausal syndrome, roots, superoxide, symptom, woman, brain, phytochemicals, symptom, perimenopause, depression, anxiety, stress, antidepressant, anxiolytic, anti-inflammatory, antioxidant, mood disorders, analgesic, Unani botanicals, MAPK, HRT, NF-κB, RNS, MCP-1, IL, catechn, cGMP, cAMP, calcium isoflavones, flavonoids, phenolic acid, tannin, protein, ROS, woman, MAO-A, Quercetin, BDNF, mitochondria, GAD, TNF-α, HPA, DHEA, DASS-21, GABA-T Thymoquinone, AMPK, anthocyanins, GSH-Px, iNOS, MDA, liquiritin

A review of PROSPERO revealed 36 records of systematic review and meta-analysis protocols focusing on interventions for menopausal and perimenopausal symptoms. Among these, 16 studies examined the use of Chinese herbal medicine and other six studies focused on acupuncture and acupoints as therapeutic approaches. One protocol specifically addressed the efficacy and safety of phytoestrogens in managing depressive disorders during perimenopause and postmenopause, employing a systematic review and meta-analysis approach. Additional protocols explored the effectiveness of herbal medicine for menopausal insomnia, with systematic reviews and meta-analyses aiming to consolidate evidence on this topic. Other research investigated the impact of non-pharmacological and alternative therapies, including their role in alleviating depression and insomnia in perimenopausal women through an overview of systematic reviews. A further protocol examined the effects of traditional East Asian medicine on symptom clusters experienced during the menopausal transition, emphasizing its holistic approach to managing these symptoms. This body of work underscores the diversity of interventions and the growing interest in evidence-based alternative and complementary therapies for menopausal health.

Moreover, computational intelligence has significantly contributed to advancements in medical diagnostics, treatment, and personalized healthcare. Technologies such as machine learning and the Internet of Things (IoT), along with methods for processing signals, text, and media, have been successfully applied in detecting sleep disorders (Heyat et al., 2015; Heyat et al., 2016; Heyat et al., 2017; Heyat et al. 2017; Guragai et al., 2020; Chola et al., 2021; Ukwuoma et al., 2022) bruxism (Heyat et al., 2019; Lai et al., 2019a; Bin Heyat et al., 2020), and cardiac diseases (Lai et al., 2019b; Lai et al., 2019c). Additionally, network visualization and word cloud techniques have been employed in analyzing women’s health conditions such as pelvic inflammatory disease (Qayyum et al., 2023; Sultana et al., 2022) [145,146], menorrhagia (Fazmiya et al., 2024), and PMS(Sultana et al., 2022; Sultana et al., 2022). These tools offer valuable insights and support in understanding and managing menopausal symptoms through advanced data-driven methodologies.

Heyat et al. conducted an extensive review of 60 articles on medical intelligence, focusing on the intersection of stressors, brain cognition, and autonomic function. Among these, 47 studies involving 37,917 participants were analyzed, alongside machine learning studies encompassing 513,767 subjects. Additionally, 13 deep learning studies explored the impact of stressors on brain health. The findings revealed that stressors contribute to increased production of catecholamines, reduced cholinergic and glucocorticoid activity, elevated cortisol levels, and persistent inflammation, collectively impairing brain function. These physiological changes were found to significantly correlate with conditions such as depression, anxiety, and cardiovascular disorders. At the cellular level, stress-induced IL-6 activation leads to elevated reactive oxygen species (ROS) and inhibition of mitochondrial cytochrome c oxidase by nitric oxide. These events intensify oxidative stress and result in mitochondrial dysfunction, characterized by oxidative damage to proteins, lipids, enzymes, and mitochondrial DNA (mtDNA). Further complications include disruptions in mitochondrial dynamics, elevated intracellular calcium levels, altered mitochondrial morphology, and neuronal death. These molecular mechanisms not only compromise brain function but also exacerbate cognitive decline. This review highlights the pressing need for multidisciplinary approaches to understand and address the complex biological and psychological pathways contributing to depression and related disorders. It emphasizes the integration of traditional therapeutic methods with modern techniques such as machine learning and deep learning to unravel the intricate interactions between oxidative stress, neuroinflammation, and mental health, paving the way for more targeted and effective interventions (Heyat et al., 2024). Weber et al.’s systematic review and meta-analysis on cognition and mood in perimenopause, highlights the heightened susceptibility to cognitive decline and the elevated risk of depressive symptoms and disorders during the menopausal transition. However, the authors concluded that findings should be interpreted with caution, as their applicability may be limited to the specific studies analyzed in the review, restricting the generalizability to broader populations or contexts (Weber et al., 2014). Madaan et al.’s short review highlights anxiety, depression, and stress are prevalent psychological issues among postmenopausal women, often overlooked due to social stigma and lack of awareness. These conditions significantly impact quality of life and warrant timely diagnosis and treatment. Reduced levels of vitamin D and estrogen, along with increased stress, are key contributors to these psychological complaints. A systematic review of case reports, observational, and cross-sectional studies highlights the need for greater awareness and proactive mental health assessments in postmenopausal women. Addressing these issues is crucial for improving both mental and physical health in this population (Acharya et al., 2021).

Shahmohammadi et al. in systematic review analyzed herbal medicine interventions for anxiety and depression in peri- and postmenopausal women. A search of trials published up to August 2017 identified 21 studies, showing that phytoestrogens significantly reduced anxiety and depression scores compared to placebo, despite high heterogeneity among trials. Notably, fennel improved symptoms in both patients with psychological issues and healthy individuals. Overall, some herbal medicines show promise in alleviating anxiety and depression in this population (Shahmohammadi et al., 2019). Badawy et al. in a systematic review and meta-analysis of seventeen cohort studies involving 16,061 women were included, with meta-analyses of seven studies (9,141 participants) using random effects models. Perimenopausal women had a significantly higher risk of depressive symptoms and diagnoses compared to premenopausal women. The findings underscore the heightened vulnerability to depression during perimenopause, though heterogeneity in menopausal classifications and depression measures may limit generalizability (Badawy et al., 2024).

A systematic reviews examined the effects of lavender on sleep quality, sexual desire, and vasomotor, psychological, and physical symptoms in menopausal and elderly women. They concluded that aromatherapy with lavender elicited feelings of relaxation, happiness, and cleanliness in over half of the participants. These findings suggest lavender as a beneficial intervention for improving various menopausal and psychological symptoms, though results should be interpreted cautiously (Roozbeh et al., 2019). A systematic review and meta-analysis concluded that lavender has significant antidepressant effects (Firoozeei et al., 2021).

Tu et al. (Tu et al., 2012) suggested that educating and communicating with parents could help reduce parental stress and anxiety. To our knowledge, these methods have been applied to menopausal symptoms, oxidative stress, and neuro-inflammation, incorporating word clouds and computational intelligence techniques.

### 3.10 Limitations, research gap and future recommendations

As this was a systematic review without a meta-analysis, effect sizes were not computed or reported. Many of the included studies used different scales, outcome measures, and study designs, which made quantitative synthesis inappropriate. This review provides a qualitative synthesis of findings rather than a pooled effect estimate.

To advance the understanding of psychological symptoms in menopausal women, it is crucial to address the need for additional data that captures the effects of these symptoms and provides comprehensive context for interpreting self-reported questionnaires. Questions requiring instinctive decisions about physical and mental health within specific criteria could offer deeper insights into the causes of menopausal symptoms. However, the limitations of self-reported measures, including the sensitivity of certain personal questions, may impact the accuracy and completeness of responses. This paper identifies such gaps and underscores the need for further research to bridge them. A larger sample size and more robust studies are necessary to substantiate the relationship between oxidative stress, neuroinflammation, depression associated with menopause, and the therapeutic potential of Unani botanicals for alleviating these psychological symptoms.

Studies, including recent findings have highlighted the potential for herb–drug interactions, particularly due to the modulation of cytochrome P450 enzymes, which may alter the metabolism of antidepressants and other medications. These interactions can lead to increased drug levels and adverse effects such as gastrointestinal disturbances and myalgia. Therefore, clinicians should carefully evaluate the risk-benefit profile and monitor patients closely when these botanicals are used alongside psychotropic drugs.

Currently, oxidative stress and neuroinflammation remain underexplored in literature, necessitating more detailed qualitative and quantitative studies with larger cohorts. Although some animal studies have demonstrated the antidepressant, anxiolytic, sedative, anti-inflammatory, and antioxidant properties of Unani botanicals, human trials are required to confirm these benefits. Further molecular and cellular-level analyses are essential to elucidate the mechanisms of action, including potential mitochondrial dysfunction linked to menopausal symptoms. Future research should prioritize identifying mitochondrial dysfunction and its cognitive implications while exploring advanced software solutions for automated detection and analysis of related phenomena. Additionally, researchers should develop more specific scales to assess menopausal psychological symptoms and establish causal connections with inflammatory markers. Evolving technologies such as brain network analysis, machine learning, quantum methods, computer vision, and blockchain technology can play a pivotal role in analyzing RCTs data, providing a sophisticated approach to understanding and addressing the complexities of menopausal psychological health. By integrating these cutting-edge technologies with the combined review methodology, future studies can better guide clinical interventions and inform policy strategies aimed at improving the wellbeing of menopausal women.

### 3.11 Applications of this review

This study distinguishes itself by providing an in-depth analysis of the pathophysiology of psychological symptoms in menopausal women, with a particular focus on the contributions of oxidative stress and neuroinflammation to depression associated with menopause. It also explores the therapeutic potential of botanicals and their mechanisms of action in alleviating these symptoms. In addition to these insights, we utilized network visualization techniques to retrieve relevant literature from the databases, offering a comprehensive overview of existing research. This work aims to benefit academicians, researchers, and scientists by highlighting the potential of artificial intelligence in efficiently retrieving data from various databases. Moreover, the identification of key related terms in our study will serve as a useful tool for researchers, students, doctors, and other academics in locating the most relevant articles in this field.

The insights derived from this study serve as a valuable resource for further advancing knowledge in the intersection of menopausal health and botanical therapeutics.

This work is unique in that it combines the methodological rigor of a systematic review with the broader scope of a scoping review. By adhering to PRISMA guidelines and utilizing systematic approaches for quality assessment and risk-of-bias evaluation, the review ensures that the findings are based on reliable evidence. At the same time, the scoping review aspect allows for a broader exploration of the various research themes, methodologies, and gaps in the field, providing a wide-ranging perspective on the subject. This combination of review types provides a more nuanced understanding of both the therapeutic potential of botanicals and the current state of research on menopausal psychological symptoms.

Furthermore, the study demonstrates the utility of computational intelligence, particularly artificial intelligence, in facilitating efficient data retrieval from various academic databases. This approach aids in identifying key terms and relevant studies, which can help researchers, students, healthcare providers, and other academics easily navigate the existing literature. The insights from this combined review approach will contribute to advancing knowledge in menopausal health and the role of botanical medicine, serving as a valuable resource for future research and clinical practice.

## 4 Conclusion

This hybrid review highlights the potential of Unani botanicals as effective natural alternatives for alleviating psychological symptoms such as depression, anxiety, and stress in menopausal women. The bioactive compounds identified in these botanicals, including Withaferin A, geranial, citronellal, geraniol, rosmarinic acid, ursolic, oleanolic acid, quercetin, caffeic acid, chlorogenic acid, rhamnocitrin, luteolin, thymoquinone, anthocyanins, stilbenes, tannins, flavonoids, alkaloids, steroids, anethole, α-pinene, cineole, borneol, camphor, thujone, ellagic acid, arabinose, galactose, and linalool, exhibit diverse pharmacological activities. These include antioxidant, anti-inflammatory, GABAA receptor agonist, MAO-inhibitory, serotonergic, sedative, antidepressant, and analgesic properties, making them promising candidates for managing menopausal psychological symptoms. To further validate their therapeutic potential, future studies should incorporate advanced methodologies, including machine learning techniques, to enhance the design and analysis of clinical trials. Rigorous research with larger sample sizes and comprehensive mechanistic evaluations is recommended to establish the safety, efficacy, and molecular actions of these bioactive molecules. This approach could pave the way for integrating Unani botanicals into evidence-based therapeutic strategies for improving the psychological wellbeing of menopausal women.

In addition, this review demonstrates the advantage of combining systematic and scoping review methodologies. By amalgamating, focused approach of a systematic review with the wider, exploratory nature of a scoping review, this hybrid methodology delivers a complete understanding of current evidence while confirming that emerging trends and knowledge gaps are acknowledged. The combination strengthens the accuracy of evidence synthesis as well as augments the context and applicability of findings across diverse studies. This method serves as an innovative model for future research, particularly in fields like botanical medicine, where diverse and often fragmented studies need to be integrated to form a coherent body of evidence. In this context, the hybrid model facilitates the identification of both specific interventions and broader research themes, fostering a holistic approach to understanding and addressing menopausal psychological health.

## Data Availability

The original contributions presented in the study are included in the article/supplementary material, further inquiries can be directed to drasnium@gmail.com, drarshiya@yahoo.com.
